# Early-life exposure to endocrine-disrupting chemicals and autistic traits in childhood and adolescence: a systematic review of epidemiological studies

**DOI:** 10.3389/fendo.2023.1184546

**Published:** 2023-06-09

**Authors:** Yandra Giovanna de Oliveira Cunha, Giovanna Cavalcanti Brito do Amaral, Alana Almeida Felix, Bruce Blumberg, Angelica Amorim Amato

**Affiliations:** ^1^ School of Medicine, University of Brasilia, Brasilia, Brazil; ^2^ Laboratory of Molecular Pharmacology, Department of Pharmaceutical Sciences, University of Brasilia, Brasilia, Brazil; ^3^ Department of Developmental and Cell Biology, University of California, Irvine, CA, United States; ^4^ Department of Pharmaceutical Sciences, University of California, Irvine, CA, United States; ^5^ Department of Biomedical Engineering, University of California, Irvine, CA, United States

**Keywords:** endocrine-disrupting chemicals (EDCs), pesticides, autism spectrum disorder, autistic traits, neurodevelopment

## Abstract

**Aims:**

Exposure to endocrine-disrupting chemicals (EDCs) during critical neurodevelopmental windows has been associated with the risk of autistic traits. This systematic review of epidemiological studies examined the association between maternal exposure to EDCs during pregnancy and the risk of autism spectrum disorder (ASD) in the offspring.

**Methods:**

We searched PubMed, Web of Science, Scopus, and Google Scholar from inception to November 17, 2022, for studies investigating the association between prenatal exposure to EDCs and outcomes related to ASD. Two independent reviewers screened studies for eligibility, extracted data, and assessed the risk of bias. The review was registered in PROSPERO (CRD42023389386).

**Results:**

We included 27 observational studies assessing prenatal exposure to phthalates (8 studies), polychlorinated biphenyls (8 studies), organophosphate pesticides (8 studies), phenols (7 studies), perfluoroalkyl substances (6 studies), organochlorine pesticides (5 studies), brominated flame retardants (3 studies), dioxins (1 study), and parabens (1 study). The number of examined children ranged from 77 to 1,556, the age at the assessment of autistic traits ranged from 3 to 14 years, and most studies assessed autistic traits using the Social Responsiveness Scale. All but one study was considered to have a low risk of bias. Overall, there was no association between maternal exposure to specific ECDs during pregnancy and the occurrence of autistic traits in offspring.

**Conclusions:**

Findings from the epidemiological studies evaluated here do not support an association between prenatal exposure to ECDs and the likelihood of autistic traits in later in life. These findings should not be interpreted as definitive evidence of the absence of neurodevelopment effects of EDCs affecting ASD risk, given the limitations of current studies such as representative exposure assessment, small sample sizes, inadequacy to assess sexually dimorphic effects, or the effects of EDC mixtures. Future studies should carefully address these limitations.

## Introduction

Autism spectrum disorder (ASD) comprises a group of heterogeneous neurodevelopmental abnormalities characterized by social communication deficits and repetitive and restrictive sensory-motor behaviors that present in a spectrum varying from very mild to severe (1). The signs and symptoms of autism typically appear early in childhood, although they may be first detected later in life when increasing social demands make limited capacities more apparent (2).

A recent systematic review of 71 ASD prevalence studies from 34 countries published over the last decade indicated a median prevalence of 1%, ranging from 0.011% to 4.36% (3). It is unclear whether the prevalence varied among different studies due to factors such as ethnicity or sociodemographic features or can be accounted for methodological differences between studies that affected the sensitivity for case finding (3). Moreover, studies conducted in the same region at different time points indicated an increase in the prevalence of ASD over time (3, 4). The latter finding most likely represents increased awareness of ASD due to various factors (3) as well as increased exposure to environmental insults that may affect neurodevelopment (5, 6). It also poses a significant concern since ASD is a leading cause of disability in children and adults (7).

The mechanisms underlying the neurodevelopmental abnormalities in ASD remain incompletely understood, despite substantial research over the past decade. The current view is that ASD is a multistage and progressive disorder of prenatal and early postnatal brain development stemming from the interplay between genetic background and environmental triggers (8). Multiple ASD risk genes have been identified, most of which are expressed prenatally in the neocortex and are involved in pathways related to excess cell proliferation and impairment of developmental processes such as neurogenesis, cell maturation, cell migration, synaptic development and function, neurofunctional activity and connectivity (9). Environmental insults may dysregulate the transcriptional programs involved in the latter pathways through epigenetic mechanisms (10).

Observational studies have examined the association between various environmental exposures at critical developmental windows and the risk of ASD. However, their findings have been inconsistent. In a recent umbrella review of published meta-analysis aimed to examine the strength and validity of reported environmental risk factors for ASD, it was found that maternal factors such as more advanced age, chronic or gestational hypertension, pre-pregnancy or gestational overweight, pre-eclampsia, pre-pregnancy antidepressant use, and selective serotonin reuptake inhibitor use during pregnancy were convincingly associated with the risk of ASD in the offspring (11).

There is also increasing evidence that prenatal exposure to environmental contaminants acting as endocrine-disrupting chemicals (EDCs) may be linked to an increased likelihood of ASD. Findings from animal models indicated that neurodevelopment is sensitive to hormone action in a time and dose-dependent manner (12). Hence, exposure to EDCs during critical windows of brain development could lead to persistent changes in behavior throughout life by various mechanisms, particularly epigenetic changes, as thoroughly reviewed elsewhere (13, 14). Data from human studies also indicated that early-life exposure to EDCs may be associated with an increased risk of neurobehavioral abnormalities later in life. This systematic review aimed to examine the current evidence from cohort and case-control studies addressing the association between prenatal exposure to EDCs and the risk of ASD.

## Methods

### Statement and registration

This systematic review and meta-analysis followed the recommendations of the Preferred Reporting Items for Systematic reviews and Meta-Analysis (PRISMA) (15) and was registered on PROSPERO (CRD42023389386).

### Search strategy

The literature search strategy was developed using the PECOS strategy, in which P (population) was defined as ‘humans,’ E (exposure) as ‘exposure to EDCs occurring during prenatal life and assessed by direct measurements of maternal biological samples,’ C (comparator), as ‘comparison group with lower or no exposure (for cohort studies) or healthy controls (for case-control studies),’ O (outcome) as ‘autistic traits or ASD assessed by validated tools,’ and S (study), as ‘observational studies.’ We searched PubMed, Web of Science, Scopus, and Google Scholar from inception to November 17, 2022, without language restriction, using the search strategy detailed in **Supplementary Table 2**. We also manually searched the reference lists of included studies and related reviews. References were managed using the Rayyan tool (16).

### Eligibility criteria, study selection, and data extraction

Two independent reviewers selected the studies (YGOC and GCBA) in two phases. First, titles and abstracts were screened from the retrieved articles to determine eligible studies. In the second phase, the full-text version of eligible studies was read by both reviewers independently to apply the inclusion criteria. Disagreements were resolved through discussion with a third reviewer (AAF).

We included studies addressing the association between prenatal exposure to EDCs and autistic traits during childhood. We excluded studies involving exposure assessment through tools other than EDC quantification in biological samples or solely assessing postnatal exposure to EDCs. Abstracts, reviews, editorials, case reports, case series, study protocols, pilot studies, animal studies, and *in vitro* studies were also excluded.

The following information was collected from the included studies: author, year of publication, type of study, EDC exposure (type of EDC, period of prenatal exposure, and method of assessment), sample size, confounding factors and covariates, age at outcome assessment, and main findings.

### Quality appraisal

The risk of bias was assessed independently by two reviewers (YGOC and GCBA) using the Joanna Briggs Institute Critical Appraisal Checklist for Cohort Studies (17), and disagreements were resolved by discussion with a third reviewer (AAA).

### Qualitative analysis

Due to differences among studies with respect to timing of exposure assessment, the method of exposure assessment, and the method of ASD or autistic traits assessment, we could not pool findings from individual studies in a meta-analysis. Therefore, we summarized data by describing results from each study in a table format (structured summary method) and by describing the effect estimates from each study based on the direction of the effect (vote counting based on the direction of effect method) (18).

## Results

**Figure 1:**
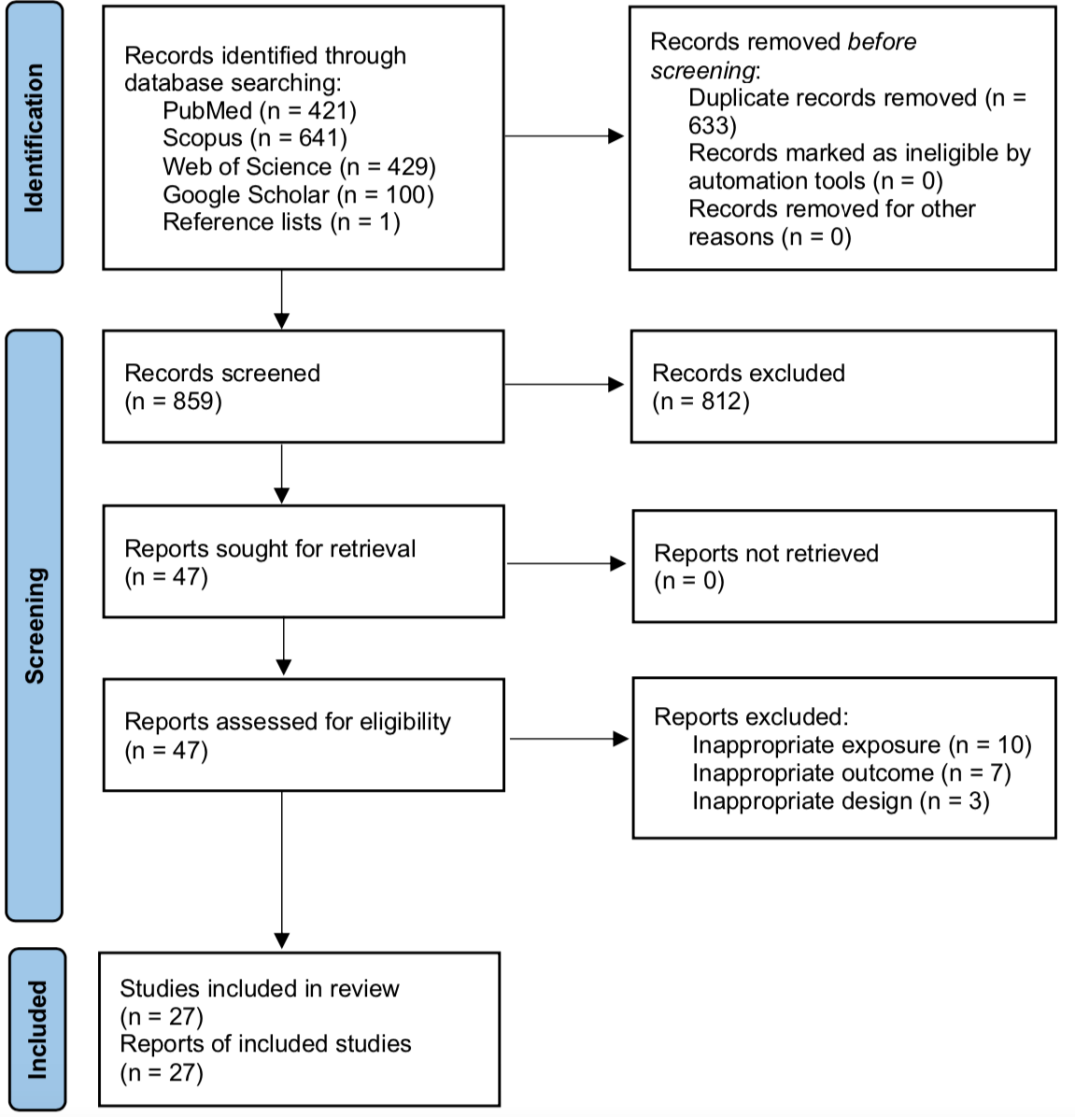
Flow diagram of literature search and study selection process.

A total of 1,491 records were retrieved through database searching and one through reference list searching. After removing duplicates, 859 records had their titles and abstracts were screened. Forty-seven records were selected, and their full texts were evaluated to determine their eligibility for inclusion. Twenty-seven studies addressed the association between maternal exposure to EDCs during pregnancy and the occurrence of autistic traits in the offspring and met our inclusion criteria (**Figure 1**). Study characteristics are described in **Supplementary Table 1** and **Supplementary Table 3**. Excluded studies with the reasons for exclusion are presented in **Supplementary Table 4**.

Twenty-one studies were cohort studies (19–25), whose participants were recruited from 13 different original cohorts. Six studies had a case-control design (25–30), of which three recruited subjects from the same population-based study (25, 29, 30). Five studies involved children from the MARBLES (Markers of Autism Risk in Babies – Learning Early Signs) Study (20, 21, 31, 32, 41), and one study investigated children from the EARLI (Early Autism Risk Longitudinal Investigation) Study (33), both cohorts of younger siblings of children with ASD. All studies were conducted in high-income countries.

Assessment of autistic traits was conducted at ages ranging from three to 14 years using validated tools, which assess social communication deficits and behavioral symptoms (restrictive and repetitive behavior), the two core impairments specific to ASD (34). The latter included the Social Responsiveness Scale (19, 22–24, 33, 35–40), Autism Diagnostic Observation Scale (20, 21, 31, 32, 41), Mullen Scales of Early Learning (20, 21, 31, 41), Diagnostic and Statistical Manual (20, 21, 25, 29–32, 41), Child Behavior Checklist (24, 42), Social Communication Questionnaire (43), Childhood Autism Spectrum Test (44), Empathy Quotient-Systemizing Quotient (37), Strengths and Difficulties Questionnaire (45), Autism Diagnostic Interview – Revised (26, 32), Behavior Assessment System for Children-2 (39), *Evaluación Neuropsicológica Infantil* (39), A Developmental NEuroPSYchological Assessment, NEPSY-II (39), and International Classification of Diseases (26–28). The Social Responsiveness Scale was the most frequently used instrument to assess autistic traits and may be administered by parents or teachers. It comprises 65 items categorized into five areas of social deficits: social awareness, social cognition, social communication, social motivation, and autistic mannerisms (46).

**Figure 2:**
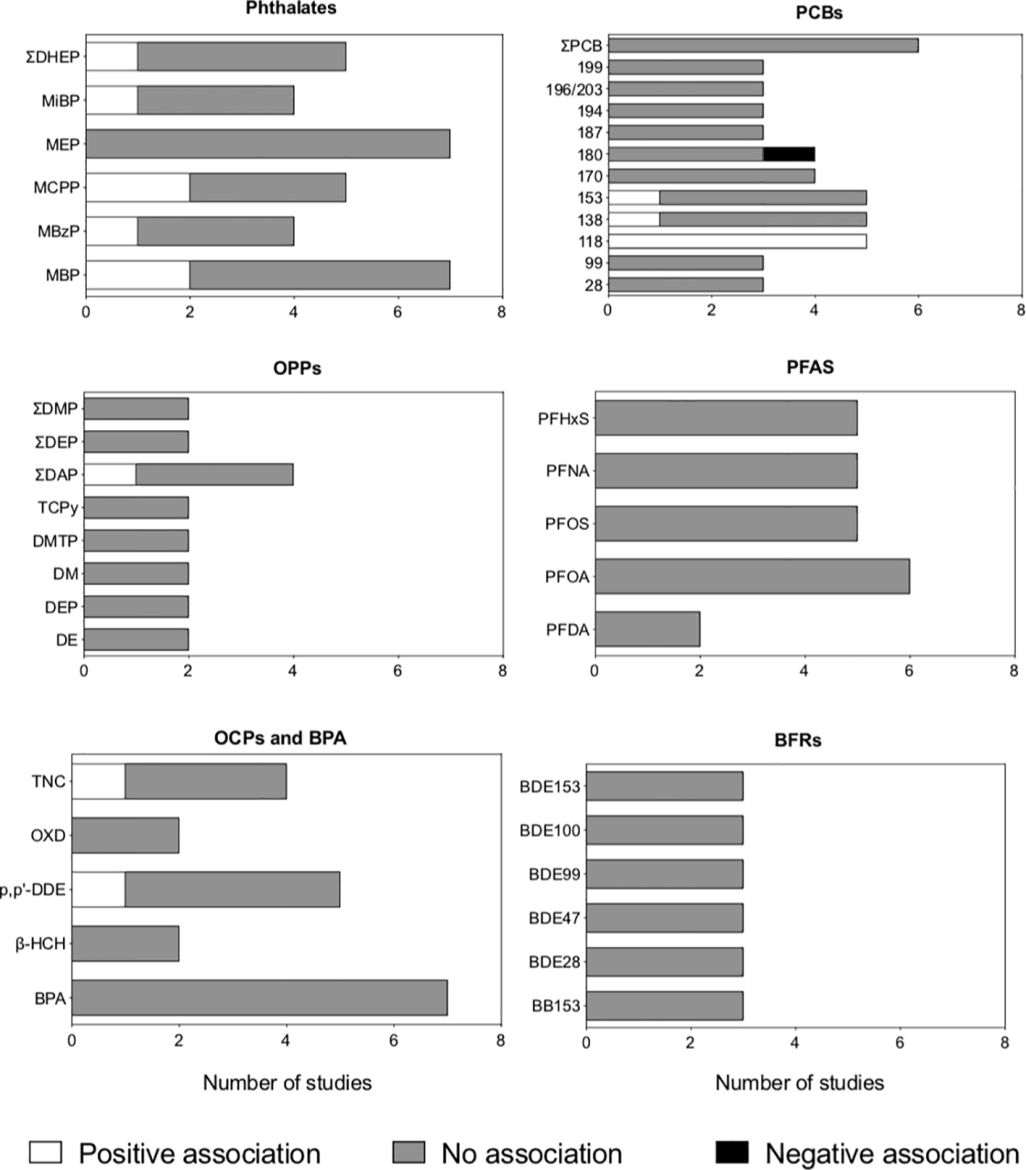
Association between antenatal exposure to endocrine-disrupting chemicals (EDCs) and autistic traits in children and adolescents, according to the type of chemical. Studies with both cross-sectional and prospective components were analyzed, and the association provided by the most conservative approach was considered (e.g., data from linear regression was considered for studies using both linear and Bayesian regression). EDCs assessed in less than three studies were not included in the figure. BFRs: brominated flame retardants; BPA, bisphenol A; OCPs, organochlorine pesticides; OPPs, organophosphate pesticides; PCBs, polybrominated biphenyls; PFAS, perfluoroalkyl substances).

Included studies assessed exposure to phthalates (19, 23, 24, 31, 33, 36, 38, 40), polychlorinated biphenyls, PCBs (19, 22, 23, 25, 26, 30, 37, 45), organophosphate pesticides, OPPs (19, 21, 32, 35, 39, 40, 44, 47), phenols (19, 20, 23, 36, 40, 42, 43), perfluoroalkyl substances, PFAS (23, 25, 27, 28, 41, 45), organochlorine pesticides, OCPs (19, 23, 25, 26, 30), brominated flame retardants (23, 25, 29), dioxins (37), and parabens (20). The results for EDCs assessed in at least three studies are summarized in **Figure 2**.

All case-control studies were considered to have a low risk of bias, whereas all but one cohort study was considered to have a low risk of bias (**Supplementary Table 5**). Due to the heterogeneous methodological design, including the tools used to assess autistic traits and the measures of association between exposure to EDCs and autistic traits, we could not summarize data quantitatively.

### Prenatal exposure to phthalates and autistic traits in childhood

Eight cohort studies examined the association between maternal exposure to phthalate metabolites during pregnancy and autistic traits in the offspring (19, 23, 24, 31, 33, 36, 38, 40). The studies examined exposure to different sets of phthalates during pregnancy; phthalate metabolites most frequently investigated were monobutyl phthalate (MBP), monoethyl phthalate (MEP), mono(3-carboxypropyl) phthalate (MCPP), and the sum of di-(2-ethyl) phthalate (DHEP) metabolites (**Supplementary Table 1**, **Figure 2**, **Supplementary Table 3**).

Two studies investigating MBP reported that antenatal exposure was positively associated with the occurrence of autistic traits in childhood (19, 38), whereas five studies reported no association (23, 24, 31, 33, 36). All seven studies examining maternal exposure to MEP during pregnancy found no association with autistic traits in the offspring (19, 23, 24, 31, 33, 36, 38). One study reported that prenatal exposure to the sum of DEHP metabolites increased the likelihood of autistic traits in childhood (33), whereas four studies examining the same exposure reported no association (19, 24, 31, 38).

One study found that exposure to the sum of DEHP metabolites during pregnancy was associated with an increased likelihood of autistic traits in the offspring (33), whereas three studies examining the same exposure found no association (19, 24, 31, 38). Two studies found that antenatal exposure to MCPP was associated with an increased likelihood of autistic traits (19, 38), whereas three studies examining the latter exposure found no association (23, 31, 33).

All but one study (36) explored differences between boys and girls. Alampi et al. (19) reported that maternal exposure to MBP, MCPP, and the sum of DEHP metabolites were associated with an increased offspring SRS scores, and MEP exposure was associated with decreased SRS scores at 3 to 4 years, and the associations were stronger for boys than girls (19). Haggerty et al. (24) reported no associations between maternal phthalate metabolites and offspring SRS scores, although stratified analysis revealed that exposure to MEP was associated with increased SRS scores in boys but not girls (24). Oulhote et al. (38) found that the associations between exposure to MCP and MCPP and SRS scores were more substantial for boys (38). Patti et al. reported that exposure to mono-isobutyl phthalate (MiBP), monobenzyl phthalate (MBzP), and the sum of DEHP metabolites and SRS scores occurred at the higher percentiles of SRS score distribution and were more robust for boys than girls (33). Conversely, Shin et al. (31) and Braun et al. (23) found no association between maternal exposure to phthalate metabolites and autistic traits in the offspring, and stratified analysis according to sex did not modify the latter findings (23, 31).

### Prenatal exposure to polychlorinated biphenyls and autistic traits in childhood

The association between maternal exposure to PCBs during pregnancy and autistic traits in the offspring was examined in five cohort studies (19, 22, 23, 37, 45) and three case-control studies (25, 26, 30), as shown in **Supplementary Table 1**, **Figure 2**, and **Supplementary Table 3**. The PCBs most frequently assessed were PCB-118, -138, and -153. One cohort study (19) and one case-control study (25) reported that higher maternal exposure to the latter PCBs was associated with an increased likelihood of autistic traits in the offspring. In contrast, five cohort studies (22, 23, 26, 37, 45) and one case-control study (25) examining the same chemicals found no association with autistic traits.

Notably, Alampi et al. (19) found no association between exposure to PCBs and autistic traits using a linear regression model. However, they identified that exposures to PCB 118, 138, 153, and 180 were associated with higher SRS scores at the upper end of the SRS score distribution using a Bayesian quantile regression model (19). Four cohort studies examined whether sex modified the associations but found no differences between boys and girls (19, 22, 23, 37).

Two case-control studies (25, 30) assessed children from the Early Markers for Autism Study using different analysis approaches. Lyall et al. (29) used a logistic regression model and found no differences in the quartiles of various maternal serum ECD levels and the odds of ASD in the offspring (30). Conversely, Hamra et al. (25) conducted a logistic regression analysis in a Bayesian framework to assess the impact of multiple EDCs within a single model. They reported that maternal exposure to PCBs was associated with an increased odds of ASD in the offspring (Hamra).

### Prenatal exposure to organophosphate pesticides and autistic traits in childhood

Eight cohort studies investigated the association between maternal exposure to OPPs and autistic traits in the offspring (19, 21, 32, 35, 39, 40, 44, 47), as shown in **Supplementary Table 1**, **Figure 2**, and **Supplementary Table 3**.

Different sets of OP metabolites were quantified during pregnancy. One study found that prenatal exposure to dialkyl phosphates was associated with an increased likelihood of autistic traits in childhood (39), whereas four studies examining the same exposure found no association with the occurrence of autistic traits in childhood (32, 35, 44, 47). In addition, the only study examining chlorpyrifos and its metabolite chlorpyrifos-oxon reported that antenatal exposure was associated with an increased likelihood of autistic traits at 11 years (44). The association between antenatal exposure to OPPs and autistic traits in childhood and adolescence was not modified by gender, as indicated by findings from six studies (19, 32, 39, 44, 47).

### Prenatal exposure to phenols and autistic traits in childhood

Seven cohort studies addressed the association between maternal exposure to phenolic compounds during pregnancy and autistic traits in the offspring (19, 20, 23, 36, 40, 42, 43), as shown in **Supplementary Table 1**, **Figure 2**, and **Supplementary Table 3**. All studies assessed exposure to bisphenol A (BPA), whereas one also examined exposure to Triclosan, TCS (19).

Overall, there was no association between exposure to BPA during pregnancy and the likelihood of autistic traits in the offspring. However, Alampi et al. (19) found that higher maternal BPA exposure was associated with an increased offspring autistic traits at the upper end of the SRS score distribution using a Bayesian quantile regression model, with no difference between boys and girls (19).

Despite not finding an overall association between prenatal BPA exposure and autistic traits, Hansen et al. (42) reported that exposure was associated with increased autistic scores at five years but not two years among children with scores above the 75th percentile, with stronger associations for girls (42). Similarly, Lim et al. (43) did not find an overall association between prenatal BPA exposure and autistic traits. However, subgroup analysis indicated that maternal exposure to BPA was associated with an increased likelihood of autistic traits in the offspring among women with higher levels of exposure and that the association was stronger for girls (43).

Alampi et al. (19) was the only study addressing exposure to TCS. The authors reported that maternal exposure to the latter EDC was not associated with an increased likelihood of autistic traits in children, although it was associated with SRS scores at the intermediate range of SRS score distribution among boys but not girls (19).

### Prenatal exposure to organochlorine pesticides and autistic traits in childhood

Exposure to OCPs was investigated in two cohort studies (19, 23), with divergent results (**Supplementary Table 1**, **Figure 2**, **Supplementary Table 3**). The most frequently investigated OCPs were p,p’-dichlorodiphenyldichloroethylene (p,p’-DDE) and *trans-*nonachlor. One study found a positive association between antenatal exposure to *trans-*nonachlor and autistic traits in the offspring (23), whereas the other found no association (19). Both studies found no association between antenatal exposure to p,p’-DDE and autistic traits in childhood (19, 23).

Interestingly, analysis considering the distribution of autistic traits scores and child gender modified the overall associations reported by some studies. Alampi et al. (19) reported no overall association between maternal exposure to β-hexachlorocyclohexane (β-HCH), p,p’-DDE, oxychlordane, or *trans*-nonachlor and the SRS score in the offspring, although Bayesian quantile regression indicated that exposure to oxychlordane and *trans*-nonachlor were associated with increased SRS scores at the intermediate range of SRS distribution among girls (19). Accordingly, Braun et al. (23) found that maternal exposure to *trans*-nonachlor was associated with higher SRS scores among girls but not boys, and the same finding was described for hexachlorobenzene exposure. The authors additionally reported that exposure to p,p’-dichloro-diphenyl-trichloroethane (p,p’-DDT) was associated with lower SRS scores, and that exposure to oxychlordane and β-HCH and p,p’-DDE was not associated with SRS scores (23).

Three case-control studies also addressed the association between prenatal exposure to OCPs and the likelihood of ASD (25, 26, 30), and only one study reported that maternal exposure to p,p’-DDE during pregnancy was associated with an increased odds of ASD in the offspring (Brown 18). The other two studies investigated subjects from the Early Markers for Autism Study but used a different approach to assess the association. Lyall et al. (29) found no differences in the quartiles of maternal serum trans-nonachlor and p,p’DDE levels and the odds of ASD in the offspring by using a logistic regression model (30). In contrast, Hamra et al. (25) conducted a logistic regression analysis in a Bayesian framework to assess the impact of multiple EDCs within a single model and found no association between maternal serum *trans-*nonachlor and p,p’DDE levels and the odds of ASD in the offspring (25).

### Prenatal exposure to other EDCs and autistic traits in childhood

Three cohorts (23, 41, 45) and three case-control (25, 27, 28) studies investigated the association between maternal exposure to different PFAS during pregnancy, either in the serum (23, 25, 27, 41, 45) or in the amniotic fluid (28). Five studies addressed PHFxS: one study found that antenatal exposure was associated with an increased likelihood of autistic traits during childhood (27), whereas four studies reported no association (25, 26, 41, 45). Five studies examined PFOS: one study found that antenatal exposure was associated with a reduced likelihood of autistic traits during childhood (28), whereas four studies reported no association (26, 27, 41, 45), as shown in **Supplementary Table 1**, **Figure 2**, and **Supplementary Table 2**. Four studies (23, 26, 28, 41) additionally stratified the results according to child gender and found it did not affect the association between prenatal exposure to PFAS and the odds of autistic traits.

One cohort (23) and two case-control (29) examined the association between prenatal exposure to brominated flame retardants and the likelihood of autistic traits in infancy (**Supplementary Table 1**, **Supplementary Table 3**). Lyall et al. (29) found that maternal exposure to PBDE-153 and the sum of PBB153 and of PBDE congeners were associated with a reduced odds of ASD in the offspring (29), whereas two studies (23, 25) examining exposure to the same brominated flame retardants found they did not affect the likelihood of autistic traits. All included studies reported no association between exposure to PBB153 and most PBDE congeners and the risk of autistic traits.

Prenatal polychlorinated dibenzo-p-furans exposure was examined in one cohort study (**Supplementary Table 1**, **Figure 2**, **Supplementary Table 3**) and was reported to be associated with decreased SRS scores in childhood among girls but not boys, and it was not related to scores from two other tools to examine autistic traits, (37). Maternal exposure to parabens was investigated in one study, and no association with the likelihood of ASD in the offspring was found (20).

## Discussion

ASD comprises a broad group of neurodevelopmental disorders sharing behavioral deficits that may be accompanied by comorbidities such as anxiety, depression, intellectual impairment, hyperactivity, motor deficits, language impairment, or self-injury (1). Despite its unknown etiology, ASD is currently viewed to result from the interplay between genetic factors and environmental influences including exposure to EDCs. This systematic review summarized findings from 27 human observational studies addressing the association between prenatal exposure to EDCs and the likelihood of autistic traits or ASD during childhood. Most included studies revealed no association between maternal exposure to EDCs during pregnancy and the odds of autistic traits or ASD in the offspring.

The findings from epidemiological studies contrast with the growing body of evidence from preclinical research indicating the biological plausibility of the association between exposure to EDCs at critical windows of neurodevelopment and subsequent risk for ASD. During early life, the developing brain is potentially exposed to EDCs by the inability of the placenta to block their transfer from the maternal circulation (48) and by the incomplete development of the blood-brain barrier to limit their entry into the central nervous system (49). In addition, the developing brain is susceptible to chemical toxicity at low concentrations that may adversely impact neurological function later in life (50). Developmental neurotoxicity may result in decreased IQ or disruption of behavior. Notably, it was estimated that the relative contribution of environmental chemicals such as organophosphate pesticides to neurodevelopmental morbidity in children was greater than that from medical conditions such as preterm birth, traumatic brain injury, brain tumors, and congenital heart disease (51). Such effects have long-known personal and economic impacts (52).

Data from several studies involving animal models have linked low-level EDC exposure to the development of autistic-like traits and provided evidence on mechanisms that could impair neurodevelopment and be related to autism. However, it should be pointed out that there is currently no widely accepted animal model of autism. A set of tests may assess animal autistic-like behavior by examining social interaction, communication, and repetitive behavior (53). Those conducted in rodents, specifically, have significantly evolved over the last decade to address the core features of ASD (54). Social interaction tests measure social responses and time spent in social interactions in pairs of animals (54). These have been the most frequently used tools to investigate autistic traits and their association with environmental chemicals in animal studies (55). Findings from animal studies indicated that the effect of early-life exposure on social behavior may vary according to experimental features such as the level, age, and duration of exposure, rodent species, sex, and the behavior test employed (14, 55).

The chemicals most explored in animal studies were pesticides, polychlorinated biphenyls, and phthalates. Chlorpyrifos is an organophosphate pesticide to which humans are ubiquitously exposed (56), and one of the most addressed pesticides in rodent studies. Gestational but not postnatal exposure of rodents to chlorpyrifos was found to lead to autistic-like behaviors later in life in studies rated as having high methodological quality in a recent systematic review (57). Male mice exposed to chlorpyrifos at levels below the threshold for observable signs of toxicity in the prenatal period exhibited decreased preference towards an unfamiliar conspecific and reduced social conditioned place preference in adulthood. This indicated disturbed conditioned and innate social behaviors and increased restricted interest (58). A second study with a similar experimental design from the same group confirmed the latter findings and suggested the sex-specific effects of early-life exposure to chlorpyrifos, given that the female offspring exhibited impaired social conditioned place preference, despite not having deficits in social preference (59).

Early-life exposure of rodents to PCBs has also been shown to phenocopy the impairment in social interactions and communication observed in ASD in humans in a concentration and sex-dependent manner (60). Perinatal exposure of male rats to PCB 47/77 mimicked the behavioral and emotional disturbances clinically observed in ASD, such as decreased behavioral flexibility and communication abilities (61). Perinatal exposure of CD1 mice to a mixture of PCBs 28, 52, 101, 138, 153, and 180 at environmentally relevant levels (10 and 1,000 ng/kg) had bidirectional effects on social behaviors. Male and female mice perinatally exposed to the PCB mixture at 10 ng/kg exhibited higher sociability, whereas males exposed to 1,000 ng/kg showed reduced social interaction with a familiar conspecific mouse, despite increasing interaction with a stranger mouse (62). More recently, Sethi et al. (63) reported that developmental mouse exposure throughout pregnancy and lactation to a PCB mixture that modeled the levels observed in pregnant women from the MARBLES cohort led to decreased sociability of male but not female offspring exposed to lower doses (63).

Exposure of female rats to a mixture of phthalates during pregnancy and lactation decreased social play behavior during puberty in the male offspring and increased time alone during social play in the female offspring when exposure was combined with a high-fat diet (64). Early-life exposure of mice to DEHP impaired their social interactions with strangers, and this was accompanied by downregulation of NMDA receptors type 1 and type 2B expression (65). More recently, Zhang et al. (66) reported that maternal exposure to DEHP during pregnancy led to impairment of sociability and induced stereotyped behavior in the male and female offspring, which were accompanied by induction of Nischarin expression in the prefrontal lobe. The authors also found that DEHP induced dendritic spine loss in primary prefrontal embryonic neurons, which was reversed by Nischarin knockdown (66).

Several programmed neurodevelopment events occur during early life and may be affected by EDC exposure, as thoroughly reviewed elsewhere (10, 14, 67). Various chemicals have been shown to disrupt hormonal pathways critical for brain development, such as thyroid hormone and sex steroid signaling. PCBs, brominated flame retardants, pesticides, PFAS, phenols, and phthalates affected various aspects of hypothalamus-pituitary-thyroid axis function (14). Similarly, pesticides, phenols, and phthalates are well-known for their ability to interfere with estrogen and androgen receptor signaling (14, 68). The latter actions may be involved in the sexually dimorphic effects of EDC exposure on neurodevelopmental outcomes.

EDCs may also induce abnormal dendritic growth, disrupt synaptic transmission by affecting neuronal calcium levels and promoting mitochondrial dysfunction, and induce abnormalities in the level, transport, and receptor expression of neurotransmitters, including gamma-aminobutyric acid, glutamate, dopamine, and serotonin (67). Moreover, EDCs can induce epigenetic changes by altering DNA methylation patterns, promoting histone modifications, and changing noncoding RNA expression, which may affect neurodevelopment in a sexually dimorphic manner (67). How these mechanisms translate into increased susceptibility to ASD remains to be understood.

The reasons for the differences in findings from human and preclinical studies are currently unknown. However, many researchers believe they may reflect the limitations in translating preclinical data into the clinical setting given that animal models do not mimic the human scenario in which there are multiple simultaneous exposures interacting with complex demographic, social, and other environmental factors that may influence the risk of ASD (14, 55). However, animal models are viewed as valuable tools to explore links between exposure to EDCs, neurodevelopment, and ASD risk, in addition to providing mechanistic information. Therefore, a better approach could be to harmonize EDCs studies in humans and animal models addressing ASD risk and then use those models to aid in understanding questions raised by human studies, such as the level and timing of exposure or even the causative chemicals in EDC mixtures (55).

Despite the no overall association between prenatal exposure to EDCs and the likelihood of ASD and autistic traits in children observed in the included epidemiological studies, we observed divergent findings for specific chemicals among the studies. The most frequently assessed EDCs in the included studies were phthalates and PCBs. Phthalate exposure was investigated in eight cohort studies (19, 23, 24, 31, 33, 36, 38, 40). Four of these reported positive associations between exposure to specific phthalate metabolites and autistic traits (19, 33, 36, 38), and one study identified negative associations (38). Among eight studies addressing exposure to PCBs (19, 22, 23, 25, 26, 30, 37, 45), only two studies reported a positive association with autistic traits (19, 30).

Many factors may account for the variable results across studies. These include differences between studies concerning population characteristics, the degree of exposure, the method of exposure and outcome assessment, the confounding factors considered, and the statistical approach used to analyze data. Although the inconsistent findings from different studies preclude a definite interpretation of the epidemiological evidence so far, they point to the need to standardize human studies to provide a better understanding of the association between exposure to EDCs and the risk of ASD.

A significant concern in exposure assessment is the possibility of misclassification of exposure based on a single or limited number of measurements for non-persistent EDCs, such as phenols, organophosphate pesticides, and phthalates (69). Pooled or serial measurements would be more accurate in assessing exposure and its hazards. Moreover, exposure assessments should ideally distinguish specific periods during development since it is currently unknown which are the sensitive periods of neurodevelopment in humans, compared to the well-established periods for experimental animals (14).

Another concern is the assessment of autistic traits in many cohort studies by tools such as the Social Responsiveness Scale. The SRS has the advantages of being strongly correlated with standard tools to diagnose ASD, such as the Autism Diagnostic Interview-Revised (70) and the Autism Diagnostic Observation Schedule (71), and of having the ability to detect subclinical abnormalities in autism-related traits (38). This could be interesting for examining more subtle effects of potential environmental insults. However, it may overestimate autistic traits in children with lower intelligence coefficients or other behavioral disorders (72, 73), which may also be influenced by EDCs (14).

Age at autistic traits assessment should also be considered, given that the effects of environmental chemicals on neurodevelopment may evolve across the life span in humans, as is observed for animal models (14). Indeed, mild cases of ASD may only be evident during adulthood in humans (74). Another major issue is taking into account the sexually dimorphic effects of EDC in neurodevelopment (14). This may also impact their association with ASD and was not addressed in six (21, 25, 29, 30, 35, 36, 45) studies included in the review.

An additional difficulty in epidemiological studies of EDC toxicity is that humans are simultaneously exposed to several chemicals, and it is not possible to accurately assess the individual effects of chemicals. Some studies included in this review accounted for multiple exposures by adjusting the impact of specific chemical exposure to exposure to other chemicals or by employing statistical methods such as the Bayesian mixture model (25) and quantile g-computation (40). Leading organizations in the field of environmental health have proposed promising strategies to address the health effects of EDC mixtures, including Bayesian Kernel machine regression, least absolute shrinkage, selection operator, and elastic net methods (75).

The relationship between EDC exposure and neurodevelopment outcomes may also be nonlinear, as observed for other outcomes of endocrine disruption (14). Moreover, susceptibility to neurodevelopmental toxicity may vary according to the presence of other risk factors, such as increased genetic susceptibility or additional insults to the developing brain. Accordingly, using a Bayesian approach, Alampi et al. (19) reported that the association between exposure to several types of EDCs and SRS scores was not uniform across SRS scores. At higher scores, indicating higher autistic behaviors, the association was stronger or weaker for some chemicals than at lower scores (19). These findings may point to specific biological features of individuals with higher SRS scores that could change their susceptibility to EDCs, which would not be evident by other methods to analyze data.

It is also important to point out that studies reporting an association between maternal exposure to EDCs and autistic traits in the offspring, either in the positive (19, 23, 26, 27, 30, 36, 38, 41) or negative (19, 26, 28, 37, 41) direction, consistently described effects of small magnitude. These observations most likely reflect the complex pathophysiological basis of autism, in which many genetic factors and environmental insults, each with a small individual effect, act in concert to determine the phenotype of most clinical forms of the disorder (1).

A limitation of our systematic review was that we could not pool the findings in a meta-analysis due to differences in study design, such as the timing of exposure assessment, the method of exposure assessment, and the method of ASD or autistic traits assessment. Moreover, all included studies were conducted in high-income countries; therefore, the findings are not readily generalizable to other settings. This is particularly important in developing countries where there is widespread exposure to EDCs that may affect neurodevelopment due to less effective control of population exposure to chemicals (76).

In conclusion, the current systematic review of epidemiological studies does not support a consistent association between prenatal exposure to individual EDCs and the likelihood of ASD or autistic traits in childhood. These findings contrast with the growing body of evidence from preclinical studies linking early-life exposure to EDC and adverse neurodevelopmental outcomes, including autistic-like behaviors. These discrepancies may be due to methodological differences between the various human studies and inherent limitations of human studies in addressing the hazards of exposure to EDCs. To aid in drawing definite conclusions on the association between EDCs and ASD, future epidemiological studies should focus on overcoming the limitations of current studies. Therefore, research should include greater geographical diversity and address different population characteristics, identify sexually dimorphic associations, improve and standardize EDC exposure measurement, identify specific susceptibility windows during neurodevelopment, reduce confounding bias, and consider the effect of EDC mixtures. In this setting, it would also be of great value if antenatal care included the assessment of exposure to ECDs by tools such as questionnaires. The latter approach could have positive impacts at the individual and populational levels by enabling healthcare providers with information to characterize and prevent exposure to EDCs, and by providing more comprehensive data for epidemiological studies on exposure to the latter chemicals and its association with health outcomes.

Almost ten years ago, Grandjean and Landrigan (50) called attention to the fact that the neurodevelopmental hazards of many chemicals, such as lead and methylmercury, were recognized only after decades of research, beginning with the effects of high-level exposure in adults, which were followed by many years of research until the risk of low-level toxicity was recognized (50). Therefore, it may take many years or even decades for the availability of definite evidence for the impact of early-life exposure to EDCs on more complex multifactorial neurodevelopmental outcomes, such as ASD, to be acknowledged. Until then, a wiser approach would be for more restrictive exposure to EDCs during critical periods of neurodevelopment.

## Data availability statement

The original contributions presented in the study are included in the article/**Supplementary Material**. Further inquiries can be directed to the corresponding author.

## Author contributions

YC, GA, and AF were responsible for the methodology, formal analysis, investigation, data curation, and writing (review and editing). AA and BB were responsible for the conceptualization, methodology, investigation, validation, data curation, and writing (original draft preparation, review and editing). All authors contributed to the article and approved the submitted version.
