# Supplementary material for: Early-life exposure to endocrine-disrupting chemicals and autistic traits in childhood and adolescence: a systematic review of epidemiological studies
Source: Front Endocrinol (Lausanne). 2023 Jun 9;14:1184546. doi: 10.3389/fendo.2023.1184546 (PMC10289191; doi:10.3389/fendo.2023.1184546)
Supplement: Supplementary file 1 [file Table_1.docx]

**Supplementary Table 1.** Summary of the characteristics of included studies (n = 27).

| **Author, year, country, study design** | **Sample size, source population** | **Type of EDC (sample), method of assessment** | **Timing of exposure** | **Outcome assessment ^1^, age at assessment** | **Adjustment confounders** | **Main findings** |
| --- | --- | --- | --- | --- | --- | --- |
| Alampi et al., 2021  Canada  Cohort | 478  MIREC study | Phthalates (MBP, MBzP, MCPP, MEP), OCPs (β-HCH, p,p’-DDE, Oxychlordane, *trans*-nonachlor) and PCBs 118, 138, 153, 180, ΣPCBs; plasma (lipid-normalized)  OPPs (DEP, DMP, DMTP), BPA, TCS; urine (specific gravity normalized)  GC/MS | 1^st^ trimester (6-13 wk) | SRS-2 (preschool-aged version, parent reported questionnaire)  3-4 y | Child’s sex, folic acid supplementation during pregnancy, caregiver environment score, household income, relationship status, maternal ethnicity, age, education, parity, and city of residence | **Phthalates**  Σ(MEHHP, MEHP, MEOHP) was associated with increased offspring SRS scores at 3-4 y  MEP exposure was associated with reduced SRS scores  The association between phthalate metabolite and SRS scores were stronger at the upper end of SRS score distribution  Associations were stronger for boys than girls  **PCBs**  Maternal exposure to PCB 118, 138, 153 was associated with increased offspring SRS scores at the upper end of SRS score distribution, at 3-4 y  No difference between boys and girls  **OPPs**  Maternal exposure to OP pesticide metabolites was not associated with changes in offspring SRS score at 3-4 y  No difference between boys and girls  **Phenols**  Maternal exposure to BPA associated with increased offspring SRS scores at the upper end of SRS score distribution, at 3-4 y, with no difference between boys and girls  Maternal exposure to TCS was not associated with increased offspring SRS scores at 3-4 y overall, although it was associated with SRS scores at the intermediate range of SRS score distribution among boys but not girls  **OCPs**  Maternal exposure to OC pesticides was not associated with offspring SRS scores at 3-4 y  Maternal exposure to oxychlordane and *trans*-nonachlor were associated with higher SRS scores at the intermediate range of SRS score distribution among girls but not boys |
| Barkoski et al., 2019  US  Cohort | 207  MARBLES Study | BPA, parabens (ETBP, MEBP, PRPB); urine (creatinine-normalized)  LC-MS/MS | 2^nd^ and 3^rd^ trimesters | ADOS, MSEL, DSM-5  3 y | Pre-pregnancy body mass index, prenatal vitamin use, homeowner status, birth year, and child’s sex | **BPA**  Maternal exposure to BPA was not associated with the risk of ASD at 3 y  No difference between boys and girls  **Parabens**  Maternal exposure to parabens was not associated with the risk of ASD at 3 y  No difference between boys and girls |
| Barkoski et al., 2021  US  Cohort | 201  MARBLES Study | 3-PBA; urine (specific gravity-normalized)  HPLC-MS/MS | 2^nd^ and 3^rd^ trimesters | MSEL, ADOS, DSM-5  3 y | Maternal pre-pregnancy BMI, homeownership, and self-reported prenatal vitamin use during the 1^st^ mo of pregnancy | Maternal exposure to 3-PBA was not associated with changes in offspring SRS score at 3 y  Gender difference not investigated |
| Bernardo et al., 2019  Canada  Cohort | 546  MIREC Study | PCB congeners (118, 138, 153, 170, 180, 187); plasma (lipid-normalized)  GC/MS | 1^st^ trimester (6-14 wk) | SRS-2 (caregiver-reported questionnaire)  3-4 y | Child’s sex, mother’s age in years, maternal race (white, other), maternal education (four levels), annual  income (four levels), marital status (married, other), ever smoked/consumed alcohol during pregnancy,  and pre-pregnancy BMI (four levels). | Maternal exposure to PCBs was not associated with changes in offspring SRS score at 3-4 y  No difference between boys and girls |
| Braun et al., 2014  US  Cohort | 172  HOME Study | PCBs (28, 66, 74, 99, 101, 105, 118, 138/158, 146, 153, 156, 157, 167, 170, 172, 177, 178, 183, 187, 194, 195, 196/203, 199, 206, 209), PFAS (PFOA, PFOS, PFHxS, PFNA), OC pesticides (β-HCH, HCB, p'p'-DDT, p'p'-DDE, oxychlordane, trans-nonachlor), BB-153, PBDEs (28, 47, 85, 99, 100, 153, 1054); serum (lipid normalized)  Phthalates (MBP, MiBP, MEP, MBzP, MCPP, MEHP, MECPP, MEHHP), BPA; urine (creatinine-normalized)  ID-GC/MS and ID-LC/MS | 2^nd^ trimester (16-26 wk) | SRS (mother-reported questionnaire)  4 and 5 y | Maternal age at delivery, race, marital status, education, parity, insurance status, employment, household income, prenatal vitamin use, depressive symptoms during the 2^nd^ trimester, maternal IQ, caregiving environment, and serum cotinine | **Phthalates**  Maternal exposure to phthalates was not associated with changes in offspring SRS score at 4-5 y  No difference between boys and girls  **PCBs**  Maternal exposure to PCBs was not associated with changes in offspring SRS score at 4-5 y  No difference between boys and girls  **BPA**  Maternal exposure to BPA was not associated with the risk of ASD at 3 y  No difference between boys and girls  **OCPs**  Maternal exposure to *trans*-nonachlor was associated with higher SRS score at 4-5 y  Maternal exposure to p’,p’-DDT was associated with lower SRS score at 4-5 y  Maternal exposure to HCB and *trans*-nonachlor was associated with higher SRS score among girls but not boys  **PBB-153 and PBDEs**  Maternal exposure to brominated flame retardants was not associated with changes in offspring SRS score at 4-5 y  No difference between boys and girls |
| Brown et al., 2018  Finland  Case–control | 778 cases of ASD and 778 control subjects  Finnish Prenatal Study of Autism, derived from the Finnish Maternity Cohort | PCBs (74, 99, 118, 138, 153, 156, 170, 180, 183, 187), OCP (p,p’-DDE); serum (lipid-normalized)  GC-MS/MS | 1^st^ and 2^nd^ trimesters (2-4 mo) | ADI-R, ICD-10  NR | Maternal age, no of previous births, socioeconomic status, maternal and parental history of psychiatric disorders, and gestational week of the blood collection | **PCBs**  Maternal exposure to PCBs was not associated with increased likelihood of ASD  No difference between boys and girls  **PFAS**  Maternal exposure to PFAS was not associated with changes in offspring SRS score at 4-5 y  No difference between boys and girls  **OCPs**  Maternal exposure to p,p’-DDE was associated with increased likelihood of ASD  The association was stronger for boys |
| Haggerty et al., 2021  USA  Cohort | 77  ARCH Study | Phthalates (MBP, MiBP, MEP, ΣDHEP; urine (creatinine-normalized)  HPLC-ESI-TQMS | 1^st^ trimester (10-14 wk) | SRS-2, CBCL (parent-reported questionnaires)  NR | Maternal BAPQ total score, maternal age at birth, pre-pregnancy BMI, prenatal education level, and prenatal household income | Maternal exposure to MEP was associated with increased offspring SRS score among boys but not girls |
| Hamra et al., 2019  USA  Case-control | 545 cases and 418 control subjects  EMA Study | PCBs (28, 99, 118, 138/158, 153, 170, 180, 187, 194, 196/203, 199), OCPs (p,p’-DDE, *trans-*nonachlor), PFAS (EtFOSAA, MeFOSAA, PHFxS, PFNA, PFOA), PBDE (28, 47, 99, 100, 153), PBB153; serum (lipid normalized)  GC-IDHRMS (PCBs, OPPs, PBDE, PBB), ID-HPLC-MS/MS (PFAS) | 2^nd^ trimester (15-20 wk) | DSM-4  4-9 y | Maternal weight, age, education, race, and child’s sex at birth | **PCBs**  Maternal exposure to PCBs was not associated with increased likelihood of ASD  **PFAS**  Maternal exposure to PFAS was not associated with increased likelihood of ASD  **OCPs**  Maternal exposure to OC pesticides was not associated with the likelihood of ASD  **PBB153 and PBDEs**  Maternal exposure to brominated flame retardants was not associated with increased likelihood of ASD  Gender difference not investigated |
| Hansen et al., 2021  Denmark  Cohort | 654 (2 y), 425 (5 y) | BPA, urine (osmolality-normalized)  LC-MS/MS | 3^rd^ trimester (26-34 wk) | DSM-oriented ASD problem scale (PDP scale),  extracted from CBCL/11⁄2-5 (parent-reported questionnaire)  2 and 5 y | Maternal education, maternal age, pre-pregnancy BMI, child’s age at assessment, parity, and child’s sex at birth | Overall maternal exposure to BPA was not associated with changes in offspring ASD score at 2 and 5 y  Maternal exposure to BPA was associated with increased ASD score at 5 y among children with ASD scores above the 75^th^ percentile, and the association was stronger for girls |
| Liew et al., 2015  Denmark  Case–control | 220 cases and 550 control subjects  The Danish National Birth Cohort | PFAS (PFOS, PFOA, PFHxS, PFHpS, PFNA, PFDA); plasma  LC-MS/MS | 1^st^ and 2^nd^ trimesters | ICD-10  NR | Maternal age at delivery, parity, socioeconomic status, maternal smoking and alcohol drinking during pregnancy, mother’s self-reported psychiatric illnesses, gestational wk of blood collection, child’s birth year, and child’s sex at birth | Maternal exposure to PFHxS was associated with increased likelihood of ASD  No difference between boys and girls |
| Lim et al., 2017  South Korea  Cohort | 413  ECD Study | BPA; urine (creatinine normalized)  HPLC-MS/MS | 2^nd^ trimester (14-27 wk) | K-SCQ (caregiver-reported questionnaire)  4 y | Parity, maternal education, child’s birth weight, child’s sex at birth, use of plastic dishes in the microwave oven, and prenatal and postnatal levels of urinary BPA | Overall maternal exposure to BPA was not associated with changes in offspring K-SCQ score at 4 y  Maternal exposure to BPA was associated with increased K-SCQ score at 4 y among women with higher BPA concentrations during pregnancy, and the association was stronger for girls |
| Lizé et al., 2022  France  Cohort | 185  French PELAGIE cohort | OPPs (DAP, DM, DE, diazinon, terbufos/metabolites, CPF/Oxon, CPF/Oxon/TCPY, TCPY); urine (creatinine-normalized)  LC/MS-MS | 1^st^ and 2^nd^ trimesters (<19 wk) | CAST (parent-reported questionnaire)  11 y | Maternal education level, maternal BMI, maternal hypertension or preeclampsia, fruit and vegetable consumption at the beginning of pregnancy, duration of breastfeeding, child’s sex at birth | Maternal exposure to chlorpyrifos and its metabolite chlorpyrifos-oxon was associated with increased incidence rate ratio of ASD at 11y  The association was stronger for boys |
| Long et al., 2019  Denmark  Case-control | 75 cases and 135 control subjects  Historic Birth Cohort | PAFS (PFOS, PFOSA, PFOA); amniotic fluid  LC-ESI-MS/MS | 2^nd^ trimester (13-28 wk) | ICD-8  NR | Birth year, child’s sex at birth, maternal age at child’s birth, paternal age at child’s birth, birth weight, gestational age at birth, gestational week at sampling, Apgar score, and parity | Maternal exposure to PFAS was associated with reduced likelihood of ASD in the offspring  No difference between boys and girls |
| Lyall et al., 2017  United States  Case-control | 545 cases and 418 control subjects  EMA Study | PCBs (28, 99, 118, 138/158, 153, 170, 180, 187, 194, 196/203, 199) and OCPs; serum (lipid-normalized)  GC-IDHRMS | 2^nd^ trimester (15-19 wk) | DSM-TR-4  NR | Child’s sex, month and year of birth, maternal age, maternal race/ethnicity, maternal weight at the time of sample collection, parity, maternal education | **PCBs**  Maternal exposure to higher levels of PCBs 138/158 and 153 were associated with increased likelihood of ASD  **OCPs**  Maternal exposure to OC pesticides was not associated the likelihood of ASD  Gender difference not investigated |
| Lyall et al., 2017  USA  Case-control study | 545 cases and 418 control subjects  EMA Study | BB153, BDEs (28, 47, 99, 100), PBDE-153; serum (lipid-normalized)  GC-IDHRMS | 2^nd^ trimester (15-20 wk) | DSM-TR-4  NR | Child’s sex, month and year of birth, maternal age, maternal race/ethnicity, maternal weight at the time of sample collection, parity, maternal education | Maternal exposure to BB-153 and the sum of BB153 and BDE congeners was associated with reduced likelihood of ASD  Gender difference not investigated |
| Millenson et al., 2017  USA  Cohort | 224  HOME Study | OPPs (DAP, DM and DE metabolites); urine (creatinine normalized)  ID-GC-MS/MS | 2^nd^ trimester (16-26 wk) | SRS (mother-reported questionnaire)  8 y | Maternal age at delivery, race, marital status, education, parity, insurance status, household income, prenatal vitamin use, maternal serum cotinine concentration, child’s sex, maternal depressive symptoms during the 2^nd^ trimester, and frequency of fresh fruit and vegetable consumption during pregnancy | Maternal exposure to DAP metabolites was not associated with changes in offspring SRS score at 8 y  Gender difference not investigated |
| Miodovnik et al., 2011  United States  Cohort | 137  Mount Sinai Children's Environmental Health Study | Phthalates (MBP, MiBP, MEP, ΣDEHP) and BPA; urine  HPLC-MS/MS | 3^rd^ trimester (25-40 wk) | SRS (mother-reported questionnaire)  7-9 y | Maternal age, maternal IQ, marital status at the time of follow-up, maternal education, child’s race, child’s sex at birth, child’s IQ, exact age at examination, and urinary creatinine | **Phthalates**  Maternal exposure to MEP and the sum of LMWP was associated increased changes in offspring SRS score at 7-9 y  **BPA**  Maternal exposure to BPA was not associated with changes in offspring SRS score at 7-9 y  Gender difference not investigated |
| Nowack et al., 2015  Germany  Cohort | 116  Duisburg Birth Cohort Study | ΣPCB (28, 52, 101, 138, 153, 180) and PCDD/Fs; serum (lipid-normalized)  HRCG/HRMS | 3^rd^ trimester (28-42 wk) | EQ-SQ, SRS (parent-reported questionnaire)  8-11 y (EQ-SQ), 9-12 y (SRS) | Child’s sex at birth, child’s age, maternal age at parturition, German nationality, maternal  education, length of gestation, older siblings, younger siblings, alcohol consumption or smoking during pregnancy, duration of breast-feeding, and maternal IQ | **PCBs**  Maternal exposure to PCBs was not associated with changes in offspring SRS, EQ, or SQ scores at 8-12 y  No difference between boys and girls  **PCDD/Fs**  Maternal exposure to PCDD/F was associated with decreased SRS score at 8-12 y, and the association was stronger for girls |
| Oh et al., 2021  US  Cohort | 173  MARBLES Study | PFAS (PFOA, PFOS, PFHxS, PFNA, PFDA, PFUnDA, PFDoDA, MeFOSAA, EtFOSAA); serum  ID-HPLC-MS/MS | 1^st^, 2^nd^, and 3^rd^ trimesters | ADOS, MSEL | Child’s sex at birth and birth year, homeownership, maternal education, and maternal vitamin intake in the 1^st^ mo of pregnancy | Maternal exposure to PFAS not was associated with the likelihood of ASD in the offspring  No difference between boys and girls |
| Oulhote et al., 2016  Denmark  Cohort | 567  General population from Faroe Islands | PCBs (138, 153, 180) and PFAS (PFOA, PFOS, PFHxS, PFNA, PFDA); serum (lipid-adjusted)  HPLC-MS/MS | 3^rd^ trimester (32 wk) | SDQ (parent-reported questionnaire)  7 y | Concurrent exposures (prenatal and postnatal exposure to PCBs and methylmercury), child’s age, child’s sex at birth, maternal age, pre-pregnancy BMI, exclusive breastfeeding duration, birth weight, parity, socio-economic status, and alcohol and smoking during pregnancy | **PCBs**  Maternal exposure to PCBs was not associated with changes in offspring SDQ scores at 7 y  **PFAS**  Maternal exposure to PFAS was not associated with changes in offspring SDQ scores at 7 y  Gender difference not investigated |
| Oulhote et al., 2020  Canada  Cohort | 556  MIREC Study. | Phthalates (MEP, MBP, MBzP, MCPP, MEHP, MEHHP, MEOHP); urine (specific gravity-normalized)  LC-MS/MS with UPLC-MS/MS | 1^st^ (6-14 wk) | SRS-2 (parent-reported questionnaire)  3-4 y | Child’s age at assessment, child’s sex at birth, birth weight, maternal age at start of pregnancy, parity, maternal education, household income, marital status, mother’s country of birth, mother’s race/ethnicity, parenting stress index, depression score, 1^st^ trimester folic acid supplementation, 1^st^ trimester blood lead concentration, alcohol consumption during pregnancy, smoking during pregnancy, city, and year of enrollment | Maternal exposure to MBP and MCPP were associated increased offspring SRS score at 3-4 y  Associations were stronger for boys than girls |
| Patti et al., 2021  US  Cohort | 140 (EARLI Study), 276 (HOME Study)  EARLI and HOME Studies | Phthalates (MCPP, MiBP, MBzP, MBP, MECCP, MEHP, MEHHP, MEOHP); urine (creatinine-normalized)  ID-HPLC/MS-MS | 1^st^, 2^nd^, and 3^rd^ trimesters (EARLI), 2^nd^ trimester (HOME) | Social Responsiveness Scale (SRS)  3 y (EARLI), 4-8 y (HOME) | Maternal age, race/ethnicity, parity, cotinine concentration, education, household income, child’s age, child’s sex at birth | Maternal exposure to MiBP, MBzP and ΣDEHP were associated increased offspring SRS score at the higher percentiles of SRS score distribution, at 3-8 y  In the HOME but not EARLI cohort, associations at the higher SRS score percentile the associations were stronger for boys than girls |
| Phillippat et al., 2018  US  Cohort | 203  MARBLES Study | OPPs (DMP, DEP, DMTP, DMDTP, DETP, DEDTP, TCPy); urine (specific gravity-normalized)  GC-MS/MS or HPLC-MS/MS | 2^nd^ and 3^rd^ trimesters | ADOS, SCQ (mother-reported questionnaire), ADI-R  3 y | Home ownership, pre-pregnancy BMI, season, date of birth | Maternal exposure to OP pesticide metabolites was not associated with changes in offspring SRS score at 3 y  No difference between boys and girls |
| Sagiv et al., 2018  US  Cohort | 247  CHAMACOS Study | OPPs (DAP metabolites DMP, DMTP, DMDTP, DEP, DETP, DEDTP); urine (creatinine-normalized)  GC-MS/MS | 1^st^ and 2^nd^ trimesters (13 wk and 26 wk) | BASC-2 (parent and teacher-reported questionnaire at 7, 10.5, 14 y), SRS-2 (parent-reported questionnaire at 14 y), ENI (9y)  NEPSI-II (12 y)  7, 9, 10.5, 12, and 14 y | Maternal age, education, country of birth, years in the United States, language of questionnaire, parity, marital status, depression, child’s age at assessment, child’s sex at birth, and quality of the home environment | Maternal exposure to OP pesticide metabolites was associated with increased autistic traits (higher SRS scores and lower BASC-2 T-score) at 7 to 14y  No difference between boys and girls |
| Shin et al., 2018  US  Cohort | 201  MARBLES Study | Phthalates (MEP, MiBP, MHiBP, MBP, MHBP, MBzP, MEHP, MEHHP, MEOHP, MECPP, MCPP, MCOP, MCNP); urine (specific gravity-normalized)  ID-HPLC-MS/MS | 2^nd^ and 3^rd^ trimesters | ADOS, MSEL  3 y | Child’s birth year, maternal pre-pregnancy BMI, homeownership as a proxy of socioeconomic status | Maternal exposure to phthalates was not associated with offspring risk of ASD at 3 y  No difference between boys and girls |
| van den Dries et al., 2019  Netherlands  Cohort | 622  Generation R Study | OPPs (DAP DEP, DMP); urine (creatinine-normalized)  GC-MS/MS | 1^st^, 2^nd^, and 3^rd^ trimesters | SRS (mother-reported questionnaire)  6 y | Maternal age, psychopathology score, ethnicity, education, income, marital status, alcohol consumption during pregnancy, non-verbal IQ, BMI, height, parity, smoking during pregnancy, and child’s sex at birth | Maternal exposure to OP pesticide metabolites was not associated with changes in offspring SRS score at 7 y  No difference between boys and girls |
| van den Dries et al., 2021  Netherlands  Cohort | 782  Generation R Study | Phthalates (MMP, MEP, MCPP, MiBP, MnBP, MECPP, MCMHP, MBzP, PA, MEOHP, MEHHP), BPA, OPPs (DMDTP, DMTP, DMP, DETP, DEP); urine (creatinine-normalized)  HPLC-ESI-MS/MS (phthalates, BPA), GC-MS/MS (OPPs). | 1^st^, 2^nd^, and 3^rd^ trimesters | SRS (mother-reported questionnaire)  7 y | Child’s sex at birth, maternal age, maternal pre-pregnancy BMI, maternal education level, maternal ethnicity, household income, marital status, parity, maternal smoking, maternal alcohol use, maternal IQ, and child age at assessment | **Phthalates**  Maternal exposure to phthalates was not associated with offspring risk of autistic traits at 6 y  No difference between boys and girls  **OPPs**  Maternal exposure to OP pesticide metabolites was not associated with changes in offspring SRS score at 7 y  No difference between boys and girls  **BPA**  Maternal exposure to BPA was not associated with offspring risk of autistic traits at 6 y  No difference between boys and girls |

^1^ Outcome assessment was conducted by the educator or health professionals, unless otherwise reported.

β-HCH: β-hexachlorocyclohexane; ADI-R: Autism Diagnostic Interview-Revised; ADOS: Autism Diagnostic Observation Schedule; ASD: Autism Spectrum Disorder; BASC-2: Behavior Assessment System for Children-2; BDE: bromodiphenyl ether; BPA: bisphenol A; BuPB: butyl paraben; CAST: Childhood Autism Spectrum Test; CBCL/1 ½ 5: Child Behaviour Checklist; 11⁄2-5; CPF: chlorpyrifos, oxon; DAP: dialkylphosphates; DE: diethylphosphates; DEP: diethylphosphate; DEPT: diethyl thiophosphate; DE: diethylphosphate metabolites (DE: DEP + DETP + DEDTP); DHEP: Di-(2-ethyl) phthalate; DM: dimethylphosphates; DMDTP: dimethyldithiophosphate; DMP: dimethylphosphate; DMTP: dimethylthiophosphate; DSM: Diagnostic and Statistical Manual; DSM-TR: Diagnostic and Statistical Manual - Text Revision; ENI: Evaluación Neuropsicológica Infantil; EQ: Empathy Quotient; Et-FOSAA: 2-(N-ethylperfluorooctane sulfonamide) acetate; ETPB: ethyl paraben; GC-IDHRMS: gas chromatography–isotope dilution high resolution mass spectrometry; GC/MS: gas chromatography coupled with mass spectrometry; GC/MS: gas chromatography coupled with tandem mass spectrometry; HCB: hexachlorbenzene; HPLC-ESI-MS/MS: high-performance liquid chromatography-electrospray ionization with tandem spectrometry; HPLC-ESI-TQMS: high-performance liquid chromatography-electrospray ionization with tandem triple quadrupole mass spectrometer; HPLC-MS/MS: high-performance liquid chromatography coupled with tandem mass spectrometry; HRCG/HRMS: capillary gas chromatography and high-resolution mass spectrometry; ICD: International Classification of diseases; ID-GC-MS/MS: isotope dilution gas chromatography coupled with tandem mass spectrometry; ID-GC/MS: isotope dilution gas chromatography coupled with mass spectrometry; ID-HPLC-MS/MS: isotope dilution high-performance liquid chromatography coupled with tandem mass spectrometry; ID-LC/MS: isotope dilution liquid chromatography coupled with mass spectrometry; K-SCQ: Korean version of the Social Communication Questionnaire; LC/MS-MS: liquid chromatography coupled with tandem mass spectrometry; MBP: monobutyl phthalate; MBzP: monobenzyl phthalate; MCMHP: mono-[(2-carboxymethyl)hexyl] phthalate; MCNP: monocarboxyl-isononly phthalate; MCOP: monocarboxyoctyl phthalate; MCPP: mono(3-carboxypropyl) phthalate; Me-FOSAA: 2-(N-methyl-perfluorooctane sulfonamide) acetate; MECPP: mono(2-ethyl-5-carboxypentyl) phthalate; MEHHP: mono(2-ethyl-5-hydroxyhexyl) phthalate; MEHP: mono(2-ethylhexyl) phthalate; MEOHP: mono(2-ethyl-5-oxohexyl) phthalate; MEP: monoethyl phthalate; MEPB: methyl paraben; MiBP: mono-isobutyl phthalate; MMP: momomethyl phthalate; MnBP: mono-n-butyl phthalate; MSEL: Mullen Scales of Early Learning; NEPSY-II: A Developmental NEuroPSYchological Assessment; OCPs: organochlorine pesticides; OPPs: organophosphate pesticides; p,p’-DDE: p,p’-dichlorodiphenyldichloroethylene; p,p’-DDT: p,p’-dichlorodiphenyltrichloroetane; PA: phthalic acid; PBA: phenoxybenzoic acid; PBB: polybrominated biphenyl; PBDE: prolibrominated diphenyl ether; PCB: polychlorinated biphenyls; PCDD/F: polychlorinated dibenzo-p-dioxins; PCDD/Fs: polychlorinated dibenzo-p-dioxins and dibenzofurans; PDP: Pervasive Developmental Problem scale; PFAS: perfluoroalkyl substances; PFDA: perfluorodecanoic acid; PFHxS: perfluorohexane sulfonic acid; PFHpS: perfluoroheptasulfonic acid; PFNA: perfluorononanoic acid; PFOA: perfluorooctanoic acid; PFOS: perfluorooctane sulfonate; PRPB: propyl paraben; SD: standard deviation; SDQ: Strength and Difficulties Questionnaire; SQ: Systemizing Quotient; SRS: Social Responsiveness Scale; TCPy: 3,5,6-trichloro-2-pyridinol; TCS: triclosan; UPLC-MS/MS: ultra-high-performance liquid chromatography coupled with tandem mass spectrometry.
