# Supplementary material for: Early-life exposure to endocrine-disrupting chemicals and autistic traits in childhood and adolescence: a systematic review of epidemiological studies
Source: Front Endocrinol (Lausanne). 2023 Jun 9;14:1184546. doi: 10.3389/fendo.2023.1184546 (PMC10289191; doi:10.3389/fendo.2023.1184546)
Supplement: Supplementary file 2 [file Table_2.docx]

**Supplementary Table 2.** Search strategies with appropriated keywords and MeSH terms.

| **Database (search date)** | **Search** |
| --- | --- |
| PubMed (November 17, 2022) | #1: “Disruptors, Endocrine” OR “Endocrine Disrupting Chemicals” OR “Chemicals, Endocrine Disrupting” OR “Endocrine Disruptor” OR “Disruptor, Endocrine” OR “Endocrine Disrupting Chemical” OR “Chemical, Endocrine Disrupting” OR “Disrupting Chemical, Endocrine” OR “Endocrine Disruptor Effect” OR “Disruptor Effect, Endocrine” OR “Effect, Endocrine Disruptor” OR “Endocrine Disruptor Effects” OR “Disruptor Effects, Endocrine” OR “Effects, Endocrine Disruptor”  #2: “Pesticides”  #3: “Agrichemical” OR “Agricultural Chemical” OR “Chemical, Agricultural” OR “Agricultural Chemicals” OR “Agrichemicals” OR “Agrochemical” OR “Chemicals, Agricultural”  #4: “Autism Spectrum Disorders” OR “Autistic Spectrum Disorder” OR “Autistic Spectrum Disorders” OR “Disorder, Autistic Spectrum”  #5: “Disorder, Autistic” OR “Disorders, Autistic” OR “Kanner's Syndrome” OR “Kanner Syndrome” OR “Kanners Syndrome” OR “Autism, Infantile” OR “Infantile Autism” OR “Autism” OR “Autism, Early Infantile” OR “Early Infantile Autism” OR “Infantile Autism, Early”  #6: (#1 OR #2 OR #3) AND (#4 OR #5) |
| Scopus (November 17, 2022) | TITLE-ABS-KEY(“Disruptors, Endocrine” OR “Endocrine Disrupting Chemicals” OR “Chemicals, Endocrine Disrupting” OR “Endocrine Disruptor” OR “Disruptor, Endocrine” OR “Endocrine Disrupting Chemical” OR “Chemical, Endocrine Disrupting” OR “Disrupting Chemical, Endocrine” OR “Endocrine Disruptor Effect” OR “Disruptor Effect, Endocrine” OR “Effect, Endocrine Disruptor” OR “Endocrine Disruptor Effects” OR “Disruptor Effects, Endocrine” OR “Effects, Endocrine Disruptor” OR “Pesticides” OR “Agrichemical” OR “Agricultural Chemical” OR “Chemical, Agricultural” OR “Agricultural Chemicals” OR “Agrichemicals” OR “Agrochemical” OR “Chemicals, Agricultural”) AND TITLE-ABS-KEY(“Autism Spectrum Disorders” OR “Autistic Spectrum Disorder” OR “Autistic Spectrum Disorders” OR “Disorder, Autistic Spectrum” OR “Disorder, Autistic” OR “Disorders, Autistic” OR “Kanner's Syndrome” OR “Kanner Syndrome” OR “Kanners Syndrome” OR “Autism, Infantile” OR “Infantile Autism” OR “Autism” OR “Autism, Early Infantile” OR “Early Infantile Autism” OR “Infantile Autism, Early”) |
| Web of Science (November 17, 2022) | TS=(“Disruptors, Endocrine” OR “Endocrine Disrupting Chemicals” OR “Chemicals, Endocrine Disrupting” OR “Endocrine Disruptor” OR “Disruptor, Endocrine” OR “Endocrine Disrupting Chemical” OR “Chemical, Endocrine Disrupting” OR “Disrupting Chemical, Endocrine” OR “Endocrine Disruptor Effect” OR “Disruptor Effect, Endocrine” OR “Effect, Endocrine Disruptor” OR “Endocrine Disruptor Effects” OR “Disruptor Effects, Endocrine” OR “Effects, Endocrine Disruptor” OR “Pesticides” OR “Agrichemical” OR “Agricultural Chemical” OR “Chemical, Agricultural” OR “Agricultural Chemicals” OR “Agrichemicals” OR “Agrochemical” OR “Chemicals, Agricultural”) AND TS=(“Autism Spectrum Disorders” OR “Autistic Spectrum Disorder” OR “Autistic Spectrum Disorders” OR “Disorder, Autistic Spectrum” OR “Disorder, Autistic” OR “Disorders, Autistic” OR “Kanner's Syndrome” OR “Kanner Syndrome” OR “Kanners Syndrome” OR “Autism, Infantile” OR “Infantile Autism” OR “Autism” OR “Autism, Early Infantile” OR “Early Infantile Autism” OR “Infantile Autism, Early”) |
| Google Scholar (November 17, 2022) | (“Endocrine disruptors” OR “endocrine disrupting chemicals” OR “pesticides” OR “agrochemicals” OR “persistent organic pollutants”) AND (“autism” OR “autism spectrum disorder”) |
