# Supplementary material for: Early-life exposure to endocrine-disrupting chemicals and autistic traits in childhood and adolescence: a systematic review of epidemiological studies
Source: Front Endocrinol (Lausanne). 2023 Jun 9;14:1184546. doi: 10.3389/fendo.2023.1184546 (PMC10289191; doi:10.3389/fendo.2023.1184546)
Supplement: Supplementary file 3 [file Table_3.docx]

**Supplementary Table 3.** Summary of the findings of included studies for the association between prenatal exposure to endocrine-disrupting chemicals and the risk of autistic traits in the offspring, according to the type of chemical.

| **Author (year), study type** | **Sample size** | **Outcome** | **Analysis** | **Findings** | **Conclusion** |
| --- | --- | --- | --- | --- | --- |
| **Phthalates (n = 8)** | | | | | |
| Alampi et al., 2021  Cohort | 478 | SRS | Linear regression (mean change in SRS score per 2-fold increase in phthalate concentrations)  Bayesian quantile regression (mean change in SRS score per 2-fold increase in phthalate concentration at the 10^th^, 30^th^, 50^th^, 70^th^, and 90^th^ percentiles of SRS score) | MBP: association with higher mean SRS scores  MBzP: no association with mean SRS scores (β coefficient reported in figure)  MCPP: association with higher mean SRS scores (β: 0.37; 95%CI: 0.08,0.67)  MEP: no association with mean SRS scores (β coefficient reported in figure)  ΣDEHP metabolites (MEHHP, MEHP, MEOHP): no association with mean SRS scores (β: 0.06; 95%CI: -0.43,0.55)  MBP: association with higher mean SRS scores, with stronger associations at higher percentiles of the SRS score distribution (β coefficient reported in figure)  MBzP: no association with mean SRS scores at any percentile of the SRS score distribution (β coefficient reported in figure)  MCPP: association with higher mean SRS scores at the 90^th^ percentile of the SRS score distribution (β: 0.79; 95%CI: 0.24,1.34)  MEP: association with lower mean SRS scores at the 50^th^, 70^th^, and 90^th^ percentiles of the SRS score distribution (β coefficient reported in figure)  ΣDEHP metabolites (MEHHP, MEHP, MEOHP): association with higher mean SRS scores at the 90^th^ percentile of the SRS score distribution (β: 0.70; 95%CI: 0.14,1.26) | Maternal exposure to MBP, MCPP, and Σ(MEHHP, MEHP, MEOHP) was associated with increased offspring SRS scores at 3-4 y  MEP exposure was associated with reduced SRS scores  The association between phthalate metabolite and SRS scores were stronger at the upper end of SRS score distribution  Associations were stronger for boys than girls |
| Braun et al., 2014  Cohort | 172 | SRS | Semi-Bayesian model (difference in SRS scores for each 2-SD increase in phthalate concentrations) | MBP: no association with SRS scores (β: -0.4; 95%CI: -2.2,1.4)  MiBP: no association with SRS scores (β: 0.3; 95%CI: -1.5,2.1)  MEP: no association with SRS scores (β: -0.5; 95%CI: -2.2, 1.3)  MBzP: no association with SRS scores (β: -0.6; 95%CI: -2.7,1.5)  MCPP: no association with SRS scores (β: 1.1; 95%CI: -0.9,3.0)  MEHP: no association with SRS scores (β: 1.4; 95%CI: -0.4,3.2)  MECPP: no association with SRS scores (β: 1.6; 95%CI: -0.3,3.4)  MEHHP: no association with SRS scores (β: 1.1; 95%CI: -0.8,3.0) | Maternal exposure to phthalates was not associated with changes in offspring SRS score at 4-5 y  No difference between boys and girls |
| Haggerty et al. 2021  Cohort | 77 | SRS and CBCL | Linear regression (SRS t-scores regressed on ln-transformed phthalate concentrations) | MBP: no association with SRS scores (β: -1.1; 95%CI: -3.0,0.9)  MiBP: no association with SRS scores (β: -1.0; 95%CI: -2.5,0.4)  MEP: no association with SRS scores (β: 0.4; 95%CI: -0.5,1.3)  ΣDEHP metabolites (MEHP, MEHHP, MEOHP, MECPP): no association with SRS scores (β: -0.5; 95%CI: -1.8,0.8) | Maternal exposure to phthalates was not associated with overall changes in offspring SRS score  Maternal exposure to MEP was associated with increased offspring SRS score among boys but not girls |
| Miodovnik et al., 2011  Cohort | 137 | SRS | Linear regression (SRS scores regressed on ln-transformed phthalate concentrations) | MBP: no association with SRS scores (β: 1.37; 95%CI: -0.43,3.17)  MEP: association with higher mean SRS scores (β: 1.38; 95%CI: 0.23,2.53)  MMP: no association with SRS scores (β: 1.29; 95%CI: -0.65,3.24)  ΣLMWP (MMP, MEP, MBP, MiBP): association with higher mean SRS scores (β: 1.53; 95%CI: 0.25,2.82)  ΣHMWP (MBzP, MPCP): no association with SRS scores (β: 0.84; 95%CI: -0.81,2.48)  ΣDEHP metabolites (MECPP, MEHHP, MEOHP, MEHP): no association with SRS scores (β: -0.83; 95%CI: -0.69,2.35) | Maternal exposure to MEP and the sum of LMWP was associated increased changes in offspring SRS score at 7-9 y  Gender difference not investigated |
| Oulhote et al., 2020  Cohort | 556 | SRS | Linear regression (adjusted mean difference in SRS score for a 2-fold increase in ln-transformed phthalate concentrations) | MBP: association with higher mean SRS scores (β: 0.6; 95%CI: 0.1,1.0)  MBzP: no association with SRS scores (β: 0.2; 95%CI: -0.2,0.6)  MEP: no association with SRS scores (β: -0.1; 95%CI: -0.4,0.1)  MCPP: association with higher mean SRS scores (β: 0.5; 95%CI: 0.1,0.8)  ΣDEHP metabolites (MEHP, MEHHP, MEOHP): no association with SRS scores (β: 0.1; 95%CI: -0.4,0.6) | Maternal exposure to MBP and MCPP were associated increased offspring SRS score at 3-4 y  Associations were stronger for boys than girls |
| Patti et al., 2021  Cohort | 140 + 276 | SRS | Linear regression (adjusted difference in SRS T-scores for a 10-fold increase in phthalate concentrations at quantiles of SRS T-scores; pooled data from EARLI and HOME studies) | MCPP: no association with SRS scores at any SRS score percentile  MiBP: association with SRS scores at the 95^th^ percentile of SRS score distribution (β: 11; 95%CI: 1,13)  MBzP: association with SRS scores at the 90^th^ and 95^th^ percentile of SRS score distribution (β: 6; 95%CI: 1,10 and β: 10; 95%CI: 6,11, respectively)  MBP: no association with SRS scores at any SRS score percentile  ΣDEHP metabolites (MECCP, MEHP, MEHHP, MEOHP): association with SRS scores at the 95^th^ percentile of SRS score distribution (β: 8; 95%CI: 3,13)  MEP: no association with SRS scores at any SRS score percentile | Maternal exposure to MiBP, MBzP and ΣDEHP were associated increased offspring SRS score at the higher percentiles of SRS score distribution, at 3-8 y  In the HOME but not EARLI cohort, associations at the higher SRS score percentile the associations were stronger for boys than girls |
| Shin et al., 2018  Cohort | 201 | ADOS; MSEL; DSM-5 | Multinomial logistic regression (relative risk ratio of ASD versus TD) | MEP: no association risk of ASD  MiBP: no association risk of ASD  MHiBP: no association risk of ASD  MBP: no association risk of ASD  MHBP: no association risk of ASD  MBzP: no association risk of ASD  MCPP: no association risk of ASD  MCOP: no association risk of ASD  MCNP: no association risk of ASD  ΣDEHP metabolites (MECCP, MEHP, MEHHP, MEOHP): no association risk of ASD | Maternal exposure to phthalates was not associated with offspring risk of ASD at 3 y  No difference between boys and girls |
| van den Dries et al., 2021  Cohort | 622 | SRS | Quantile-based g-computation method | Phthalate metabolite mixture (MMP, MEP, MCPP, MiBP, MnBP, MECPP, MCMHP, MBzP, PA, MEOHP, MEHHP): no association with autistic traits Difference in adjusted mean SRS:  Q1 ref  Q2: -0.02 (95%CI -0.04,0.01)  Q3: -0.03 (95%CI -0.09,0.02)  Q4: -0.05 (95%CI -0.13,0.03) | Maternal exposure to phthalates was not associated with offspring risk of autistic traits at 6 y  No difference between boys and girls |
| **Polychlorinated biphenyls (n = 8)** | | | | | |
| Alampi et al., 2021  Cohort | 478 | SRS | Linear regression  Bayesian quantile regression for each 2-fold increases in PCB concentration | PCB 118: no association with mean SRS scores (β coefficient reported in figure)  PCB 138: no association with mean SRS scores (β coefficient reported in figure)  PCB 153: no association with mean SRS scores (β coefficient reported in figure)  PCB 180: no association with mean SRS scores (β coefficient reported in figure)  ΣPCB (180, 138, 153, 180): no association with mean SRS scores (β coefficient reported in figure)  PCB 118: positive association at the higher percentiles of the SRS score distribution (β coefficient reported in figure)  PCB 138: positive association at the higher percentiles of the SRS score distribution (β coefficient reported in figure)  PCB 153: positive association at the higher percentiles of the SRS score distribution (β coefficient reported in figure)  PCB 180: positive association at the higher percentiles of the SRS score distribution (β coefficient reported in figure)  ΣPCB (180, 138, 153, 180): positive association at the higher percentiles of the SRS score distribution (β coefficient reported in figure) | Maternal exposure to PCB 118, 138, 153 was associated with increased offspring SRS scores at the upper end of SRS score distribution, at 3-4 y  No difference between boys and girls |
| Bernardo et al., 2019  Cohort | 546 | SRS | Bayesian linear regression  Bayesian predictive odds ratio | SRS adjusted mean score (95%CI) vs Q1  PCB 118: Q1 0.0; Q2: 0.09 (−1.46, 1.63); Q3: -0.02 (−1.55, 1.53); Q4: 0.26 (−1.34, 1.88)  PCB 138: Q1: 0.0; Q2: 0.70 (−0.63, 2.04); Q3: 0.44 (−1.11, 2.01); Q4: 1.35 (−0.42, 3.16)  PCB 153: Q1 0.0; Q2: 0.58 (−1.02, 2.19); Q3: −0.50 (−2.25, 1.26); Q4: 1.10 (−0.71, 2.89)  PCB 170: Q1 0.0; Q2: −0.33 (−1.66, 1.02); Q3: −0.14 (−1.64, 1.33); Q4: 0.83 (−0.97, 2.62)  PCB 180: Q1 0.0; **Q2: −1.57 (−2.93, −0.16)**; Q3: −1.13 (−2.75, 0.50); Q4: 0.19 (−1.60, 1.97)  PCB 187: Q1 0.0; Q2: −0.49 (−1.83, 0.88); Q3: −0.46 (−1.84, 0.94); Q4: 0.51 (−1.15, 2.15)  ΣPCB: Q1 0.0; Q2: 0.60 (−0.75, 1.96); Q3: 0.67 (−1.21, 2.53); Q4: 1.45 (−0.98, 3.90)  OR (95%CI) of SRS > 60, vs Q1  PCB 118: Q1 1.0; Q2: 0.93 (0.57, 1.44); Q3: 1.0 (0.62, 1.53); Q4: 1.20 (0.72, 1.89)  PCB 138: Q1: 1.0; Q2: 1.21 (0.79, 1.76); Q3: 1.36 (0.84, 2.09); Q4: 1.76 (0.99, 2.92)  PCB 153: Q1 1.0; Q2: 1.36 (0.80, 2.16); Q3: 1.09 (0.62, 1.78); Q4: 1.82 (1.02, 3.02)  PCB 170: Q1 1.0; Q2 0.90 (0.60, 1.31); Q3: 1.04 (0.65, 1.58); Q4: 1.39 (0.80, 2.24)  PCB 180: Q1 1.0; Q2 0.63 (0.40, 0.96); Q3: 0.79 (0.46, 1.24); Q4: 1.20 (0.67, 1.98)  PCB 187: Q1 1.0; Q2 0.92 (0.60, 1.34); Q3: 0.99 (0.65, 1.44); Q4: 1.46 (0.89, 2.24)  ΣPCB: Q1 1.0; Q2 1.32 (0.88, 1.92); Q3: 1.44 (0.82, 3.77); Q4: 1.97 (0.90, 3.77) | Maternal exposure to PCBs was not associated with changes in offspring SRS score at 3-4 y  No difference between boys and girls |
| Braun et al., 2014  Cohort | 172 | SRS | Semi-Bayesian model (difference in SRS scores for each 2-SD increase in PCB concentrations)  Semi-Bayesian model (difference in SRS scores for detectable vs non-detectable PCB) | PCB 28: no association with SRS scores (β: 1.0; 95%CI: -0.9, 2.8)  PCB 74: no association with SRS scores (β: -0.6; 95%CI: -2.7, 1.6)  PCB 99: no association with SRS scores (β: 0.0; 95%CI: -2.1, 2.1)  PCB 105: no association with SRS scores (β: 0.5; 95%CI: -1.7, 2.6)  PCB 118: no association with SRS scores (β: -0.4; 95%CI: -2.4, 1.6)  PCB 138/158: no association with SRS scores (β: -0.2; 95%CI: -2.5, 2.1)  PCB 146: no association with SRS scores (β: -0.9; 95%CI: -3.5, 1.6)  PCB 153: no association with SRS scores (β: -1.5; 95%CI: -4.1, 1.2)  PCB 156: no association with SRS scores (β: -1.2; 95%CI: -4.0, 1.6)  PCB 170: no association with SRS scores (β: -1.3; 95%CI: -4.2, 1.7)  PCB 183: no association with SRS scores (β: -0.2; 95%CI: -2.3, 2.0)  PCB 187: no association with SRS scores (β: -1.3; 95%CI: -3.5, 0.9)  PCB 194: no association with SRS scores (β: -2.5; 95%CI: -4.9, 0.0)  PCB 196/203: no association with SRS scores (β: 0.0; 95%CI: -2.5, 2.6)  PCB 199: no association with SRS scores (β: -1.1; 95%CI: -3.7, 1.5)  PCB 206: no association with SRS scores (β: 0.7; 95%CI: -1.8, 3.1)  PCB 209: no association with SRS scores (β: 0.5; 95%CI: -1.6, 2.7)  PCB 66: no association with SRS scores (β: 0.9; 95%CI: -1.4, 3.3)  PCB 101: no association with SRS scores (β: -0.2; 95%CI: -2.1, 1.8)  PCB 157: no association with SRS scores (β: -0.5; 95%CI: -2.6, 1.7)  PCB 167: no association with SRS scores (β: 0.3; 95%CI: -1.8, 2.5)  PCB 172: no association with SRS scores (β: -1.3; 95%CI: -3.4, 0.9)  PCB 177: no association with SRS scores (β: -0.5; 95%CI: -2.5, 1.4)  PCB 178: no association with SRS scores (β: -2.6; 95%CI: -4.7, -0.4)  PCB 195: no association with SRS scores (β: -0.2; 95%CI: -2.3, 1.9) | Maternal exposure to PCBs was not associated with changes in offspring SRS score at 4-5 y  No difference between boys and girls |
| Brown et al., 2018  Cohort | 778 (ASD), 778 (controls) | ICD-10, ADI-Revised | Logistic regression (change in the odds of ASD for PCB concentration > p75) | ΣPCBs (74, 99, 118, 138, 153, 156, 170, 180, 183, 187): no association with the likelihood of ASD (OR 0.95; 95%CI 0.73-1.24) | Maternal exposure to PCBs was not associated with increased likelihood of ASD  No difference between boys and girls |
| Nowack et al., 2015  Cohort | 116 | SRS, EQ, SQ | Hierarchical linear regression (SRS, EQ, and SQ scores and PCB concentrations) | SRS  ΣPCBs (28, 52, 101, 138, 153, 180): no association with SRS scores (β: -3.99; 95%CI: -8.61, 0.64)  EQ  ΣPCBs (28, 52, 101, 138, 153, 180): no association with SRS scores (β: 0.74; 95%CI: -2.81, 4.30)  SQ  ΣPCBs (28, 52, 101, 138, 153, 180): no association with SRS scores (β: 0.92; 95%CI: -2.24, 4.09) | Maternal exposure to PCBs was not associated with changes in offspring SRS, EQ, or SQ scores at 8-12 y  No difference between boys and girls |
| Oulhote et al., 2016  Cohort | 567 | SDQ | Linear regression (SDQ score per 2-fold increase in PCB concentrations) | ΣPCBs (138, 153, 180): no association with SRS scores (β: -0.28; 95%CI: -0.65, 0.10) | Maternal exposure to PCBs was not associated with changes in offspring SDQ scores at 7 y  Gender difference not investigated |
| Hamra et al., 2019  Case-control | 545 (ASD), 418 (controls) | DSM-4 | Logistic regression in a Bayesian framework (change in the odds of ASD per one standard deviation increase in the z-score of PCB concentrations) | Mixture prior  PCB 28: no association with the likelihood of ASD (OR 1.00; 95%CI 0.96-1.04)  PCB 99: no association with the likelihood of ASD (OR 0.99; 95%CI 0.96-1.03)  PCB 118: no association with the likelihood of ASD (OR 0.99; 95%CI 0.96-1.03)  PCB 138/158: no association with the likelihood of ASD (OR 0.99; 95%CI 0.96-1.03)  PCB 153: no association with the likelihood of ASD (OR 0.99; 95%CI 0.96-1.03)  PCB 170: no association with the likelihood of ASD (OR 0.99; 95%CI 0.96-1.04)  PCB 180: no association with the likelihood of ASD (OR 0.99; 95%CI 0.96-1.04)  PCB 187: no association with the likelihood of ASD (OR 0.99; 95%CI 0.96-1.03)  PCB 194: no association with the likelihood of ASD (OR 0.99; 95%CI 0.96-1.04)  PCB 196/203: no association with the likelihood of ASD (OR 0.99; 95%CI 0.96-1.03)  PCB 199: no association with the likelihood of ASD (OR 0.99; 95%CI 0.96-1.03)  Shared mean  PCB 28: no association with the likelihood of ASD (OR 1.00; 95%CI 0.95-1.10)  PCB 99: no association with the likelihood of ASD (OR 0.99; 95%CI 0.93-1.07)  PCB 118: no association with the likelihood of ASD (OR 0.99; 95%CI 0.93-1.07)  PCB 138/158: no association with the likelihood of ASD (OR 0.99; 95%CI 0.93-1.08)  PCB 153: no association with the likelihood of ASD (OR 0.99; 95%CI 0.92-1.07)  PCB 170: no association with the likelihood of ASD (OR 1.00; 95%CI 0.93-1.08)  PCB 180: no association with the likelihood of ASD (OR 1.00; 95%CI 0.93-1.09)  PCB 187: no association with the likelihood of ASD (OR 0.99; 95%CI 0.92-1.07)  PCB 194: no association with the likelihood of ASD (OR 0.99; 95%CI 0.93-1.08)  PCB 196/203: no association with the likelihood of ASD (OR 0.99; 95%CI 0.92-1.07)  PCB 199: no association with the likelihood of ASD (OR 0.99; 95%CI 0.93-1.08) | Maternal exposure to PCBs was not associated with increased likelihood of ASD  Gender difference not investigated |
| Lyall et al., 2017  Case-control | 545 (ASD), 418 (controls) | DSM-4-TR | Logistic regression (odds of ASD according to the quartile of PCB concentrations) | PCB 28: no association with the likelihood of ASD (Q1 OR 1; Q2 OR 1.17, 95%CI 0.79-1.73; Q3 OR 1.20, 95%CI 0.81-1.77; Q4 OR 1.27, 95%CI 0.87, 1.86)  PCB 99: no association with the likelihood of ASD (Q1 OR 1; Q2 OR 1.12, 95%CI 0.74-1.70; Q3 OR 1.08, 95%CI 0.72-1.62; Q4 OR 1.17, 95%CI 0.75, 1.83)  PCB 118: no association with the likelihood of ASD (Q1 OR 1; Q2 OR 1.29, 95%CI 0.86-1.95; Q3 OR 1.38, 95%CI 0.90-2.11; Q4 OR 1.15, 95%CI 0.72, 1.82)  PCB 138/158: higher exposure associated with increased likelihood of ASD (Q1 OR 1; Q2 OR 1.39, 95%CI 0.92-2.10; Q3 OR 1.34, 95%CI 0.87-2.07; Q4 OR 1.79, 95%CI 1.10, 2.92)  PCB 153: higher exposure associated with increased likelihood of ASD (Q1 OR 1; Q2 OR 1.32, 95%CI 0.88-1.99; Q3 OR 1.24, 95%CI 0.80-1.93; Q4 OR 1.82, 95%CI 1.10, 3.02)  PCB 170: no association with the likelihood of ASD (Q1 OR 1; Q2 OR 1.15, 95%CI 0.76-1.76; Q3 OR 1.17, 95%CI 0.75-1.83; Q4 OR 1.48, 95%CI 0.88, 2.50)  PCB 180: no association with the likelihood of ASD (Q1 OR 1; Q2 OR 1.00, 95%CI 0.66-1.50; Q3 OR 1.17, 95%CI 0.75-1.81; Q4 OR 1.49, 95%CI 0.89, 2.49)  PCB 187: no association with the likelihood of ASD (Q1 OR 1; Q2 OR 0.89, 95%CI 0.58-1.36; Q3 OR 1.22, 95%CI 0.79-1.87; Q4 OR 1.32, 95%CI 0.79, 2.20)  PCB 194: no association with the likelihood of ASD (Q1 OR 1; Q2 OR 0.99, 95%CI 0.65-1.49; Q3 OR 1.06, 95%CI 0.68-1.63; Q4 OR 1.24, 95%CI 0.76, 2.03)  PCB 196/203: no association with the likelihood of ASD (Q1 OR 1; Q2 OR 0.79, 95%CI 0.52-1.21; Q3 OR 1.05, 95%CI 0.69-1.61; Q4 OR 1.13, 95%CI 0.68, 1.88)  PCB 199: no association with the likelihood of ASD (Q1 OR 1; Q2 OR 0.93, 95%CI 0.61-1.41; Q3 OR 1.17, 95%CI 0.76-1.80; Q4 OR 1.13, 95%CI 0.68, 1.89)  ΣPCBs (above PCBs): no association with the likelihood of ASD (Q1 OR 1; Q2 OR 1.08, 95%CI 0.72-1.63; Q3 OR 0.99, 95%CI 0.64-1.51; Q4 OR 1.36, 95%CI 0.88, 2.11) | Maternal exposure to higher levels of PCBs 138/158 and 153 were associated with increased likelihood of ASD  Gender difference not investigated |
| **Organophosphate pesticides (n = 8)** | | | | | |
| Alampi et al., 2014  Cohort | 478 | SRS | Linear regression (mean change in SRS score per 2-fold increase in OP pesticide metabolite concentrations)  Bayesian quantile regression (mean change in SRS score per 2-fold increase in OP pesticide metabolite concentrations at the 10^th^, 30^th^, 50^th^, 70^th^, and 90^th^ percentiles of SRS score) | DEP: no association with mean SRS scores (β coefficient reported in figure)  DMP: no association with mean SRS scores (β coefficient reported in figure)  DMTP: no association with mean SRS scores (β coefficient reported in figure)  DEP: no association with mean SRS scores at any percentile of the SRS score distribution (β coefficient reported in figure)  DMP: no association with mean SRS scores at any percentile of the SRS score distribution (β coefficient reported in figure)  DMTP: no association with mean SRS scores at any percentile of the SRS score distribution (β coefficient reported in figure) | Maternal exposure to OP pesticide metabolites was not associated with changes in offspring SRS score at 3-4 y  No difference between boys and girls |
| Barkoski et al., 2021  Cohort | 201 | ADOS, MSEL, DSM-5 | Logistic regression (change in the relative risk ratio of ASD vs TD for a 1-unit change in the ln-transformed 3-PBA concentration) | 3-PBA: no association with the likelihood of ASD (RRR 1.34; 95%CI 0.89-2.03) | Maternal exposure to 3-PBA was not associated with changes in offspring SRS score at 3 y  Gender difference not investigated |
| Lizé et al., 2022  Cohort | 185 | CAST (Childhood Autism Spectrum Test) | Binomial regression (incidence rate ratio of ASD according to the tercile of OP pesticide/metabolite concentration) | DAP: no association with the incidence rate ratio of ASD (T1: IRR 1.0; T2: IRR 0.95, 95%CI 0.77-1.18; T3 IRR 0.93, 95% CI 0.75-1.15; continuous β: 1.01; 95%CI: 0.75, 1.36)  DM: no association with the incidence rate ratio of ASD (T1: IRR 1.0; T2: IRR 0.90, 95%CI 0.73-1.11; T3 IRR 0.91, 95% CI 0.74-1.12; continuous β: 0.75; 95%CI: 0.49, 1.12)  DE: no association with the incidence rate ratio of ASD (Non-detectable: IRR 1.0; detectable, below median: IRR 0.86, 95%CI 0.69-1.06; detectable, above median: IRR 0.93, 95% CI 0.75-1.14)  Diazinon: no association with the incidence rate ratio of ASD (Non-detectable: IRR 1.0; detectable, below median: IRR 1.03, 95%CI 0.80-1.31; detectable, above median: IRR 1.25, 95% CI 0.99-1.57)  Terbufos/metabolites: no association with the incidence rate ratio of ASD (Non-detectable: IRR 1.0; detectable: IRR 1.03, 95% CI 0.84-1.25)  CPF/Oxon: association with increased incidence rate ratio of ASD (Non-detectable: IRR 1.0; detectable: IRR 1.29, 95% CI 1.07-1.57)  CPF/Oxon/TCPY: no association with the incidence rate ratio of ASD (Non-detectable: IRR 1.0; detectable: IRR 1.18, 95% CI 0.98-1.43)  TCPY: no association with the incidence rate ratio of ASD (Non-detectable: IRR 1.0; detectable: IRR 1.00, 95% CI 0.74-1.35) | Maternal exposure to chlorpyrifos and its metabolite chlorpyrifos-oxon was associated with increased incidence rate ratio of ASD at 11y  The association was stronger for boys |
| Millenson et al., 2017  Cohort | 224 | SRS | Logistic regression (change in SRS score for a 10-fold in the sum of DAP metabolite concentrations) | ΣDAP: no association with the SRS score (β: -1.2; 95%CI: -4.0, 1.6)  DM: no association with the SRS score (β: -0.8; 95%CI: -3.3, 1.6)  DE: no association with the SRS score (β: -0.8; 95%CI: -2.9, 1.2) | Maternal exposure to DAP metabolites was not associated with changes in offspring SRS score at 8 y  Gender difference not investigated |
| Phillippat et al., 2018  Cohort | 148 | ADOS, ADI-R | Logistic regression (change in the odds of ASD for a doubling of OP pesticide metabolite concentrations) | TCPy: no association with the likelihood of ASD (OR 0.80; 95%CI 0.57-1.12)  DMTP: no association with the likelihood of ASD (OR 0.99; 95%CI 0.79-1.25)  DEP: no association with the likelihood of ASD (OR 0.94; 95%CI 0.74-1.20)  ΣDMP: no association with the likelihood of ASD (OR 0.92; 95%CI 0.73-1.15)  ΣDEP: no association with the likelihood of ASD (OR 0.91; 95%CI 0.72-1.16)  ΣDAP: no association with the likelihood of ASD (OR 0.89; 95%CI 0.68-1.17) | Maternal exposure to OP pesticide metabolites was not associated with changes in offspring SRS score at 3 y  No difference between boys and girls |
| Sagiv et al., 2018  Cohort | 247 | SRS (at 14 y), BASC-2 (at 7, 10.5, 14 y), ENI (at 9 y), NEPSYII (at 12 y) | Linear regression (change in SRS score for a 10-fold increase in OP pesticide metabolite concentration) | SRS  ΣDAPs: associated with higher SRS scores (β 2.7; 95%CI 0.9, 4.5)  ΣDEs (DEP, DEPT, DMDTP): no association with SRS scores (β 1.0; 95%CI -0.8, 2.7)  ΣDM (DMP, DMTP, DMDTP): associated with higher SRS scores (β 2.4; 95%CI 0.8, 4.0)  BASC-2 T-score (7y, teacher assessment)  ΣDAPs: associated with lower BASC-2 T-score (β -2.9; 95%CI -5.4, -0.4)  ΣDEs (DEP, DEPT, DMDTP): no association with BASC-2 T-score (β 0.0; 95%CI -2.2, 2.3)  ΣDM (DMP, DMTP, DMDTP): associated with lower BASC-2 T-score (β -3.0; 95%CI -5.2, -0.8)  BASC-2 T-score (7, 10.5, 14 y, parent assessment)  ΣDAPs: associated with lower BASC-2 T-score (β -3.1; 95%CI -4.9, -1.2)  ΣDEs (DEP, DEPT, DMDTP): no association with BASC-2 T-score (β -0.5; 95%CI -2.3, 1.4)  ΣDM (DMP, DMTP, DMDTP): associated with lower BASC-2 T-score (β -2.8; 95%CI -4.4, -1.2)  ENI (9y)  ΣDAPs: no association with ENI scores (β -0.1; 95%CI -0.3, 0.2)  ΣDEs (DEP, DEPT, DMDTP): no association with ENI scores (β -0.1; 95%CI -0.4, 0.2)  ΣDM (DMP, DMTP, DMDTP): no association with ENI scores (β -0.1; 95%CI -0.5, 0.3)  NESPY-II (12y)  ΣDAPs: no association with NESPY scores (β 0.4; 95%CI -0.5, 1.3)  ΣDEs (DEP, DEPT, DMDTP): no association with NESPY scores (β 0.6; 95%CI -0.2, 1.4)  ΣDM (DMP, DMTP, DMDTP): no association with NESPY scores (β 0.2; 95%CI -0.6, 1.0) | Maternal exposure to OP pesticide metabolites was associated with increased autistic traits (higher SRS scores and lower BASC-2 T-score) at 7 to 14y  No difference between boys and girls |
| van den Dries et al., 2019  Cohort | 622 | SRS | Linear regression (difference in autistic traits score per 10-fold increase in OP pesticide concentration) | Total DAP  < 18 wk: no association with SRS scores (β: 0.15; 95%CI: -0.11, 0.40)  18-25 wk: no association with SRS scores (β: -0.03; 95%CI: -0.32, 0.26)  > 25 wk: no association with SRS scores (β: 0.05; 95%CI: -0.22, 0.32)  DEP  < 18 wk: no association with SRS scores (β: 0.04; 95%CI: -0.13, 0.21)  18-25 wk: no association with SRS scores (β: -0.02; 95%CI: -0.21, 0.16)  > 25 wk: no association with SRS scores (β: -0.16; 95%CI: -0.37, 0.05)  DMP  < 18 wk: no association with SRS scores (β: 0.16; 95%CI: -0.08, 0.40)  18-25 wk: no association with SRS scores (β: -0.03; 95%CI: -0.30, 0.25)  > 25 wk: no association with SRS scores (β: 0.12; 95%CI: -0.15, 0.38) | Maternal exposure to OP pesticide metabolites was not associated with changes in offspring SRS score at 7 y  No difference between boys and girls |
| van den Dries et al., 2021  Cohort | 782 | SRS | Quantile-based g-computation method | OP pesticide metabolites (DMDTP, DMTP, DMP, DETP, DEP): no association with autistic traits (difference in adjusted mean SRS relative to the first quantile, Q1: Q2: 0.00, 95%CI -0.03,0.02; Q3: -0.01, 95%CI -0.05,0.04; Q4: -0.01, 95%CI -0.08,0.06) | Maternal exposure to OP pesticide metabolites was not associated with changes in offspring SRS score at 7 y  No difference between boys and girls |
| **Phenols (n = 7)** | | | | | |
| Alampi et al., 2021  Cohort | 478 | SRS | Linear regression (mean change in SRS score per 2-fold increase in phenol concentrations)  Bayesian quantile regression (mean change in SRS score per 2-fold increase in phenol concentration at the 10^th^, 30^th^, 50^th^, 70^th^, and 90^th^ percentiles of SRS score) | BPA: no association with mean SRS scores (β coefficient reported in figure)  TCS: no association with mean SRS scores (β coefficient reported in figure)  BPA: association with higher mean SRS scores at the higher percentiles of the SRS score distribution (β coefficient reported in figure)  TCS: no association with mean SRS scores at any percentile of the SRS score distribution (β coefficient reported in figure), although TCS concentrations were associated with higher SRS scores at the 30^th^, 50^th^, and 70^th^ percentiles of the SRS score distribution among boys (β coefficient reported in figure) | Maternal exposure to BPA associated with increased offspring SRS scores at the upper end of SRS score distribution, at 3-4 y, with no difference between boys and girls  Maternal exposure to TCS was not associated with increased offspring SRS scores at 3-4 y overall, although it was associated with SRS scores at the intermediate range of SRS score distribution among boys but not girls |
| Barkoski et al., 2019  Cohort | 201 | ADOS, MSEL, DSM-5 | Logistic regression (change in the relative risk ratio of ASD vs TD for a 1-unit change in the ln-transformed BPA concentration or one quartile change in chemical concentration) | BPA  Average pregnancy 2^nd^ and 3^rd^ trimester exposure:  ln-transformed concentration: OR 0.59 (95%CI 0.36-0.96)  Quartiled concentration: OR 0.91 (95%CI 0.91-1.27)  BPA  Average pregnancy 2^nd^ trimester exposure:  ln-transformed concentration: OR 0.75 (95%CI 0.45-1.23)  Quartiled concentration: OR 0.72 (95%CI 0.48-1.09)  BPA  Average pregnancy 3^rd^ trimester exposure:  ln-transformed concentration: OR 0.66 (95%CI 0.37-1.18)  Quartiled concentration: OR 0.95 (95%CI 0.67-1.34) | Maternal exposure to BPA was not associated with the risk of ASD at 3 y  No difference between boys and girls |
| Braun et al., 2014  Cohort | 172 | SRS | Linear regression (change in the odds of ASD per one ln-transformed BPA concentration) | No association with SRS scores (β coefficient reported in figure) | Maternal exposure to BPA was not associated with the risk of ASD at 3 y  No difference between boys and girls |
| Hansen et al., 2021  Cohort | 654/425 | ASD score from PDP scale extracted from the CBCL/ 1 ½-5) | Negative binomial regression (OR of relative change in ASD-score)  Logistic regression (OR of ASD score > p75 and > p90 across the terciles of BPA concentration)  Logistic regression (OR of ASD score > p75 and > p90 across continuous ln-transformed BPA concentration) | BPA  ASD score at 2 y (n = 654)  1^st^ tercile: OR 1.0  2^nd^ tercile: OR 0.87 (95%CI 0.71-1.07)  3^rd^ tercile: OR 1.02 (95%CI 0.84-1.24)  ASD score at 5 y (n = 425)  1^st^ tercile: OR 1.0  2^nd^ tercile: OR 1.13 (95%CI 0.89-1.42)  3^rd^ tercile: OR 1.23 (95%CI 0.98-1.53)  ASD score > p75 at 2 y (n = 166)  1^st^ tercile: OR 1.0  2^nd^ tercile: OR 0.81 (95%CI 0.47-1.20)  3^rd^ tercile: OR 1.06 (95%CI 0.70-1.68)  ASD score > p90 at 2 y (n = 98)  1^st^ tercile: OR 1.0  2^nd^ tercile: OR 0.84 (95%CI 0.47-1.48)  3^rd^ tercile: OR 1.18 (95%CI 0.69-2.00)  ASD score > p75 at 5 y (n = 86)  1^st^ tercile: OR 1.0  2^nd^ tercile: OR 1.22 (95%CI 0.63-2.36)  3^rd^ tercile: OR 1.80 (95%CI 0.97-3.32)  ASD score > p90 at 5 y (n = 59)  1^st^ tercile: OR 1.0  2^nd^ tercile: OR 0.86 (95%CI 0.36-1.99)  3^rd^ tercile: OR 1.65 (95%CI 0.78-3.49)  ASD score > p75 at 2 y (n = 166)  OR 1.08 (95%CI 0.94-1.24)  ASD score > p90 at 2 y (n = 98)  OR 1.10 (95%CI 0.93-1.31)  ASD score > p75 at 5 y (n = 86)  OR 1.25 (95%CI 1.03-1.49)  ASD score > p90 at 5 y (n = 59)  OR 1.20 (95%CI 0.94-1.53) | Overall maternal exposure to BPA was not associated with changes in offspring ASD score at 2 and 5 y  Maternal exposure to BPA was associated with increased ASD score at 5 y among children with ASD scores above the 75^th^ percentile, and the association was stronger for girls |
| Lim et al., 2017  Cohort | 413 | K-SCQ | Linear regression (percent change in K-SCQ score associated with 2-fold increases in BPA concentrations) | BPA  Overall  +3.4% (95%CI -0.8,7.7), p = 0.2464  BPA concentration < 3.0 μg/g creatinine  -0.1 (95%CI -4.8,4.9)  BPA concentration > 3.0 μg/g creatinine  16.9 (95%CI 2.3,33.5) | Overall maternal exposure to BPA was not associated with changes in offspring K-SCQ score at 4 y  Maternal exposure to BPA was associated with increased K-SCQ score at 4 y among women with higher BPA concentrations during pregnancy, and the association was stronger for girls |
| Miodovnik et al., 2011  Cohort | 137 | SRS | Linear regression (SRS scores regressed on ln-transformed phthalate concentrations) | BPA: no association with SRS scores (β: 1.18; 95%CI: -0.75,3.11) | Maternal exposure to BPA was not associated with changes in offspring SRS score at 7-9 y  Gender difference not investigated |
| van den Dries et al., 2021  Cohort | 622 | SRS | Quantile-based g-computation method | BPA: no association with autistic traits Difference in adjusted mean SRS:  Q1 ref  Q2: 0.00 (95%CI -0.02,0.01)  Q3: -0.01 (95%CI -0.04,0.02)  Q4: -0.01 (95%CI -0.06,0.04) | Maternal exposure to BPA was not associated with offspring risk of autistic traits at 6 y  No difference between boys and girls |
| **Perfluoroalkyl substances (n = 6)** | | | | | |
| Braun et al., 2014  Cohort | 172 | SRS | Semi-Bayesian model (difference in SRS scores for each 2-SD increase in PFAS concentrations) | PFOA: no association with SRS scores (β: -2.0; 95%CI: -4.4,0.4)  PFOS: no association with SRS scores (β coefficient reported in figure)  PFNA: no association with SRS scores (β coefficient reported in figure)  PFHxS: no association with SRS scores (β coefficient reported in figure) | Maternal exposure to PFAS was not associated with changes in offspring SRS score at 4-5 y  No difference between boys and girls |
| Liew et al., 2015  Case–control | 220 (ASD), 550 (controls) | ICD-10 | Linear regression (change in the odds of ASD per one ln-transformed PFAS concentration)  Logistic regression (odds of ASD according to the quartile of PFAS concentrations) | PFOS: no association with the likelihood of ASD (OR 1.21; 95%CI 0.69-2.13)  PFOA: no association with the likelihood of ASD (OR 1.15; 95%CI 0.68-1.93)  PFHxS: no association with the likelihood of ASD (OR 1.26; 95%CI 1.00-1.58)  PFNA: no association with the likelihood of ASD (OR 0.84; 95%CI 0.48-1.49)  PFHpS: no association with the likelihood of ASD (OR 0.82; 95%CI 0.56-1.22)  PFDA: no association with the likelihood of ASD (OR 0.82; 95%CI 0.53-1.28)  PFOS: no association with the likelihood of ASD  Q1: OR 1.00  Q2: OR 1.05 (95%CI 0.73-1.50)  Q3: OR 1.20 (95%CI 0.77-1.89)  Q4: OR 1.16 (95%CI 0.65-2.09)  PFOA: no association with the likelihood of ASD  Q1: OR 1.00  Q2: OR 1.11 (95%CI 0.76-1.60)  Q3: OR 0.97 (95%CI 0.63-1.48)  Q4: OR 0.93 (95%CI 0.54-1.59)  PFHxS: associated with increased likelihood of ASD at intermediate exposure levels  Q1: OR 1.00  Q2: OR 1.55 (95%CI 1.06-2.28)  Q3: OR 1.86 (95%CI 1.25-2.76)  Q4: OR 1.33 (95%CI 0.84-2.11)  PFNA: no association with the likelihood of ASD  Q1: OR 1.00  Q2: OR 0.94 (95%CI 0.66-1.34)  Q3: OR 0.73 (95%CI 0.49-1.08)  Q4: OR 0.98 (95%CI 0.59-1.63)  PFHpS: no association with the likelihood of ASD  Q1: OR 1.00  Q2: OR 0.70 (95%CI 0.49-1.01)  Q3: OR 0.83 (95%CI 0.53-1.31)  Q4: OR 0.80 (95%CI 0.44-1.48)  PFDA: no association with the likelihood of ASD  Q1: OR 1.00  Q2: OR 0.99 (95%CI 0.72-1.37)  Q3: OR 1.34 (95%CI 0.92-1.95)  Q4: OR 0.73 (95%CI 0.43-1.24) | Maternal exposure to PFHxS was associated with increased likelihood of ASD  No difference between boys and girls |
| Oulhote et al., 2016  Cohort | 567 | SDQ | Linear regression (SDQ score per 2-fold increase in PFAS concentrations)  Linear regression (adjusted mean difference in SDQ score per 2-fold increase in PFAS concentrations) | ΣPFASs (PFOA, PFOS, PFHxS, PFNA, PFDA): no association with SRS scores (β: 0.86; 95%CI: -1.54, 3.25)  PFOA: -0.37 (95%CI -1.34,0.61)  PFOS: 0.46 (95%CI -0.78,1.7)  PFHxS: -0.28 (95%CI -0.75,0.18)  PFNA: -0.11 (95%CI -1.2,0.98)  PFDA: -0.01 (95%CI -0.98,0.96) | Maternal exposure to PFAS was not associated with changes in offspring SDQ scores at 7 y  Gender difference not investigated |
| Hamra et al., 2019  Case-control | 545 (ASD), 418 (controls) | DSM-4 | Logistic regression in a Bayesian framework (change in the odds of ASD per one standard deviation increase in the z-score of PFAS concentrations) | Mixture prior  EtFOSAA: no association with the likelihood of ASD (OR 0.99; 95%CI 0.95-1.03)  MeFOSAA: no association with the likelihood of ASD (OR 0.99; 95%CI 0.96-1.04)  PFHxS: no association with the likelihood of ASD (OR 1.00; 95%CI 0.96-1.05)  PFNA: no association with the likelihood of ASD (OR 0.99; 95%CI 0.94-1.03)  PFOA: no association with the likelihood of ASD (OR 0.99; 95%CI 0.96-1.03)  Shared mean  EtFOSAA: no association with the likelihood of ASD (OR 0.99; 95%CI 0.91-1.05)  MeFOSAA: no association with the likelihood of ASD (OR 1.00; 95%CI 0.95-1.09)  PFHxS: no association with the likelihood of ASD (OR 1.00; 95%CI 0.95-1.10)  PFNA: no association with the likelihood of ASD (OR 0.98; 95%CI 0.90-1.04)  PFOA: no association with the likelihood of ASD (OR 0.99; 95%CI 0.93-1.06) | Maternal exposure to PFAS was not associated with increased likelihood of ASD  Gender difference not investigated |
| Long et al., 2019  Case-control | 75 (ASD), 135 (controls) | ICD-8 | Unconditional logistic regression | PFOS: OR 0.41 (95%CI 0.17-0.97, p = 0.042)  PFOSA: OR 0.92 (95%CI 0.79-1.07, p = 0.272)  PFOA: OR 0.16 (95%CI 0.01-2.13, p = 0.167)  ΣPFSA: OR 0.90 (95%CI 0.77-1.05, p = 0.178)  ΣPFCA: OR 0.15 (95%CI 0.01-1.76, p = 0.131)  ΣPFSA+ΣPFCA: OR 0.90 (95%CI 0.77-1.04, p = 0.158) | Maternal exposure to PFAS was associated with reduced likelihood of ASD in the offspring  No difference between boys and girls |
| Oh et al., 2021  Cohort | 173 | ADOS, MSEL, DSM-5 | Poisson regression model (relative risk of ASD compared with typical development per 2-fold increase in log-2 transformed PFAS concentrations)  Poisson regression model (relative risk of ASD compared with typical development per each 2 ng/mL increase in PFAS concentrations) | Log 2-transforned PFAS concentration  PFOA: no association with the likelihood of ASD (RR 1.2, 95%CI 0.90-1.61)  PFOS: no association with the likelihood of ASD (RR 0.97, 95%CI 0.74-1.28)  PFHxS: no association with the likelihood of ASD (RR 0.88, 95%CI 0.77-1.01)  PFNA: no association with the likelihood of ASD (RR 1.24, 95%CI 0.91-1.69)  Untransformed PFAS concentration  PFOA: no association with the likelihood of ASD (RR 1.31, 95%CI 1.04-1.65)  PFOS: no association with the likelihood of ASD (RR 0.99, 95%CI 0.90-1.08)  PFHxS: no association with the likelihood of ASD (RR 0.90, 95%CI 0.54-1.51)  PFNA: no association with the likelihood of ASD (RR 1.79, 95%CI 1.13-2.85) | Maternal exposure to PFAS not was associated with the likelihood of ASD in the offspring  No difference between boys and girls |
| **Organochlorine pesticides (n = 5)** | | | | | |
| Alampi et al., 2021  Cohort | 478 | SRS | Linear regression (mean change in SRS score per 2-fold increase in OC pesticide concentrations)  Bayesian quantile regression (mean change in SRS score per 2-fold increase in OC pesticide concentration at the 10^th^, 30^th^, 50^th^, 70^th^, and 90^th^ percentiles of SRS score) | β-HCH: no association with mean SRS scores (β coefficient reported in figure)  p,p’-DDE: no association with mean SRS scores (β coefficient reported in figure)  Oxychlordane: no association with mean SRS scores (β coefficient reported in figure)  *trans*-nonachlor: no association with mean SRS scores (β coefficient reported in figure)  β-HCH: no association with mean SRS scores at any percentile of the SRS score distribution (β coefficient reported in figure)  p,p’-DDE: no association with mean SRS scores at any percentile of the SRS score distribution (β coefficient reported in figure)  Oxychlordane: no association with mean SRS scores at any percentile of the SRS score distribution (β coefficient reported in figure)  *trans*-nonachlor: no association with mean SRS scores at any percentile of the SRS score distribution (β coefficient reported in figure) | Maternal exposure to OC pesticides was not associated with offspring SRS scores at 3-4 y  Maternal exposure to oxychlordane and *trans*-nonachlor were associated with higher SRS scores at the intermediate range of SRS score distribution among girls but not boys |
| Braun et al., 2014  Cohort | 172 | SRS | Semi-Bayesian model (difference in SRS scores for each 2-SD increase in OC pesticide concentrations)  Semi-Bayesian model (difference in SRS scores for detectable vs non-detectable OC pesticide) | HCB: no association with SRS scores  p,p’-DDE: no association with SRS scores  Oxychlordane: no association with SRS scores  *trans*-nonachlor: association with higher SRS scores (β 4.1; 95%CI 0.8, 7.3)  β-HCH: no association with SRS scores  p,p’-DDT: association with lower SRS scores (β -3.3; 95%CI -6.1, -0.5) | Maternal exposure to *trans*-nonachlor was associated with higher SRS score at 4-5 y  Maternal exposure to p’,p’-DDT was associated with lower SRS score at 4-5 y  Maternal exposure to HCB and *trans*-nonachlor was associated with higher SRS score among girls but not boys |
| Brown et al., 2018  Case–control | 778 (ASD), 778 (controls) | ICD-10, ADI-Revised | Logistic regression (change in the odds of ASD for OC pesticide concentration > p75) | p,p’-DDE: associated with increased likelihood of ASD (OR 1.32; 95%CI 1.02-1.71) | Maternal exposure to p,p’-DDE was associated with increased likelihood of ASD  The association was stronger for boys |
| Hamra et al., 2019  Case-control | 545 (ASD), 418 (controls) | DSM-4 | Logistic regression in a Bayesian framework (change in the odds of ASD per one standard deviation increase in the z-score of OC pesticide metabolite concentrations) | Mixture prior  p,p’-DDE: no association with the likelihood of ASD (OR 0.99; 95%CI 0.92-1.02)  *trans*-nonachlor: no association with the likelihood of ASD (OR 0.99; 95%CI 0.96-1.03)  Shared mean  p,p’-DDE: no association with the likelihood of ASD (OR 0.98; 95%CI 0.89-1.03)  *trans*-nonachlor: no association with the likelihood of ASD (OR 1.00; 95%CI 0.94-1.07) | Maternal exposure to OC pesticides was not associated with the likelihood of ASD  Gender difference not investigated |
| Lyall et al., 2017  Case-control | 545 (ASD), 418 (controls) | DSM-4 | Logistic regression (odds of ASD according to the quartile of OC pesticide concentrations) | p,p’-DDE: no association with the likelihood of ASD  Q1: OR 1  Q2: OR 1.35 (95%CI 0.93-1.96)  Q3: OR 1.16 (95%CI 0.77-1.74)  Q4: OR 0.90 (95%CI 0.57-1.42)  *trans*-nonachlor: no association with the likelihood of ASD  Q1: OR 1  Q2: OR 0.92 (95%CI 0.62-1.36)  Q3: OR 0.84 (95%CI 0.56-1.26)  Q4: OR 0.84 (95%CI 0.55-1.28) | Maternal exposure to OC pesticides was not associated the likelihood of ASD  Gender difference not investigated |
| **Brominated flame retardants (n = 3)** | | | | | |
| Braun et al., 2014  Cohort | 172 | SRS | Semi-Bayesian model (difference in SRS scores for each 2-SD increase in brominated flame retardant concentrations)  Semi-Bayesian model (difference in SRS scores for detectable vs non-detectable brominated flame retardant) | PBB-153: no association with SRS scores (β coefficient reported in figure)  PBDE-28: no association with SRS scores (β 2.5; 95%CI -0.6,5.6)  PBDE-47: no association with SRS scores (β coefficient reported in figure)  PBDE-99: no association with SRS scores (β coefficient reported in figure)  PBDE-100: no association with SRS scores (β coefficient reported in figure)  PBDE-153: no association with SRS scores (β coefficient reported in figure)  PBDE-85: association with lower SRS scores (β -3.1; 95%CI -5.9, -0.5)  PBDE-154: no association with SRS scores (β coefficient reported in figure) | Maternal exposure to brominated flame retardants was not associated with changes in offspring SRS score at 4-5 y  No difference between boys and girls |
| Hamra et al., 2019  Case-control | 545 (ASD), 418 (controls) | DSM-4 | Logistic regression in a Bayesian framework (change in the odds of ASD per one standard deviation increase in the z-score of brominated flame retardant concentrations) | Mixture prior  PBB153: no association with the likelihood of ASD (OR 0.99; 95%CI 0.96-1.02)  PBDE-28: no association with the likelihood of ASD (OR 0.99; 95%CI 0.96-1.03)  PBDE-47: no association with the likelihood of ASD (OR 0.99; 95%CI 0.95-1.03)  PBDE-99: no association with the likelihood of ASD (OR 0.99; 95%CI 0.96-1.03)  PBDE-100: no association with the likelihood of ASD (OR 0.99; 95%CI 0.96-1.03)  PBDE-153: no association with the likelihood of ASD (OR 0.99; 95%CI 0.87-1.01)  Shared mean  PBB153: no association with the likelihood of ASD (OR 0.99; 95%CI 0.92-1.05)  PBDE-28: no association with the likelihood of ASD (OR 0.99; 95%CI 0.92-1.06)  PBDE-47: no association with the likelihood of ASD (OR 0.99; 95%CI 0.91-1.06)  PBDE-99: no association with the likelihood of ASD (OR 0.99; 95%CI 0.92-1.06)  PBDE-100: no association with the likelihood of ASD (OR 0.99; 95%CI 0.92-1.06)  PBDE-153: no association with the likelihood of ASD (OR 0.98; 95%CI 0.88-1.03) | Maternal exposure to brominated flame retardants was not associated with increased likelihood of ASD  Gender difference not investigated |
| Lyall et al., 2017  Case-control | 545 (ASD), 418 (controls) | DSM-4-TR | Logistic regression (odds of ASD according to the quartile of brominated flame retardant concentrations) | PBB153: no association with the likelihood of ASD (Q1 OR 1; Q2 OR 1.14, 95%CI 0.76-1.70; Q3 OR 0.86, 95%CI 0.56-1.31; Q4 OR 1.12, 95%CI 0.73, 1.71)  BDE-28: no association with the likelihood of ASD (Q1 OR 1; Q2 OR 0.95, 95%CI 0.65-1.39; Q3 OR 0.89, 95%CI 0.60-1.31; Q4 OR 0.82, 95%CI 0.56, 1.22)  BDE-47: no association with the likelihood of ASD (Q1 OR 1; Q2 OR 0.77, 95%CI 0.53-1.10; Q3 OR 0.71, 95%CI 0.49-1.02; Q4 OR 0.73, 95%CI 0.50, 1.05)  BDE-99: no association with the likelihood of ASD (Q1 OR 1; Q2 OR 0.75, 95%CI 0.52-1.08; Q3 OR 0.72, 95%CI 0.49-1.04; Q4 OR 0.70, 95%CI 0.48, 1.01)  BDE-100: no association with the likelihood of ASD (Q1 OR 1; Q2 OR 0.66, 95%CI 0.45-0.95; Q3 OR 0.68, 95%CI 0.47-0.98; Q4 OR 0.69, 95%CI 0.47, 1.00)  PBDE-153: associated with decreased likelihood of ASD (Q1 OR 1; Q2 OR 0.62, 95%CI 0.43-0.90; Q3 OR 0.71, 95%CI 0.49-1.03; Q4 OR 0.56, 95%CI 0.38, 0.84)  ΣBB153 and BDE congeners: associated with decreased likelihood of ASD (Q1 OR 1; Q2 OR 0.83, 95%CI 0.58-1.18; Q3 OR 1.01, 95%CI 0.71-1.44; Q4 OR 0.64, 95%CI 0.44, 0.93) | Maternal exposure to BDE-53 and the sum of BB153 and BDE congeners was associated with reduced likelihood of ASD  Gender difference not investigated |
| **Dioxins (n = 1)** | | | | | |
| Nowack et al. 2015  Cohort | 116 | SRS, EQ, SQ | Hierarchical linear regression (SRS, EQ, and SQ scores and PCDD/F concentrations) | SRS  PCDD/F: associated with decreased SRS scores (β: -6.66; 95%CI: -11.88, -1.44)  EQ  PCDD/F: no association with SRS scores (β: 2.21; 95%CI: -1.63, 6.05)  SQ  PCDD/F: no association with SRS scores (β: 1.43; 95%CI: -2.02, 4.87) | Maternal exposure to PCDD/F was associated with decreased SRS score at 8-12 y, and the association was stronger for girls |
| **Parabens (n = 1)** | | | | | |
| Barkoski et al., 2019  Cohort | 201 | ADOS, MSEL, DSM-5 | Logistic regression (change in the relative risk ratio of ASD vs TD for a 1-unit change in the ln-transformed paraben concentration or one quartile change in chemical concentration) | ETPB  Average pregnancy 2^nd^ and 3^rd^ trimester exposure:  ln-transformed concentration: OR 1.23 (95%CI 0.99-1.53)  Quartiled concentration: OR 1.32 (95%CI 0.94-1.87)  Average pregnancy 2^nd^trimester exposure:  ln-transformed concentration: OR 1.31 (95%CI 1.00-1.71)  Quartiled concentration: OR 1.38 (95%CI 0.91-2.11)  Average pregnancy 3^rd^ trimester exposure:  ln-transformed concentration: OR 1.23 (95%CI 0.97-1.58)  Quartiled concentration: OR 1.36 (95%CI 0.95-1.95)  MEPB  Average pregnancy 2^nd^ and 3^rd^ trimester exposure:  ln-transformed concentration: OR 0.94 (95%CI 0.76-1.17)  Quartiled concentration: OR 1.08 (95%CI 0.78-1.51)  Average pregnancy 2^nd^ trimester exposure:  ln-transformed concentration: OR 0.88 (95%CI 0.66-1.18)  Quartiled concentration: OR 0.96 (95%CI 0.64-1.45)  Average pregnancy 3^rd^ trimester exposure:  ln-transformed concentration: OR 1.07 (95%CI 0.86-1.35)  Quartiled concentration: OR 1.12 (95%CI 0.80-1.59)  PRPB  Average pregnancy 2^nd^ and 3^rd^ trimester exposure:  ln-transformed concentration: OR 1.12 (95%CI 0.89-1.41)  Quartiled concentration: OR 1.18 (95%CI 0.84-1.65)  P  Average pregnancy 2^nd^ trimester exposure:  ln-transformed concentration: OR 1.09 (95%CI 0.82-1.46)  Quartiled concentration: OR 0.91 (95%CI 0.70-1.17)  Average pregnancy 3^rd^ trimester exposure:  ln-transformed concentration: OR 1.13 (95%CI 0.92-1.40)  Quartiled concentration: OR 1.20 (95%CI 0.85-1.71) | Maternal exposure to parabens was not associated with the risk of ASD at 3 y  No difference between boys and girls |

95%CI: 95% confindence interval; β-HCH, β-hexachlorocyclohexane; ADI-R: Autism Diagnostic Interview-Revised; ADOS: Autism Diagnostic Observation Schedule; ASD: Autism Spectrum Disorder; BASC-2: Behavior Assessment System for Children-2; BDE: bromodiphenyl ether; BPA: bisphenol A; BuPB: butyl paraben; CAST: Childhood Autism Spectrum Test; CBCL/1 ½ 5: Child Behaviour Checklist; 11⁄2-5; CPF: chlorpyrifos, oxon; DAP: dialkylphosphates; DE: diethylphosphates; DEP: diethylphosphate; DEPT: diethyl thiophosphate; DE: diethylphosphate metabolites (DE: DEP + DETP + DEDTP); DHEP: Di-(2-ethyl) phthalate; DM: dimethylphosphates; DMDTP: dimethyldithiophosphate; DMP: dimethylphosphate; DMTP: dimethylthiophosphate; DSM: Diagnostic and Statistical Manual; DSM-TR: Diagnostic and Statistical Manual - Text Revision; ENI: *Evaluación Neuropsicológica Infantil;* EQ: Empathy Quotient; Et-FOSAA: 2-(N-ethylperfluorooctane sulfonamide) acetate; ETPB: ethyl paraben; HCB: hexachlorbenzene; ICD: International Classification of diseases; IRR: incidence risk ratio; K-SCQ: Korean version of the Social Communication Questionnaire; MBP: monobutyl phthalate; MBzP: monobenzyl phthalate; MCMHP: mono-[(2-carboxymethyl)hexyl] phthalate; MCNP: monocarboxyl-isononly phthalate; MCOP: monocarboxyoctyl phthalate; MCPP: mono(3-carboxypropyl) phthalate; Me-FOSAA: 2-(N-methyl-perfluorooctane sulfonamide) acetate; MECPP: mono(2-ethyl-5-carboxypentyl) phthalate; MEHHP: mono(2-ethyl-5-hydroxyhexyl) phthalate; MEHP: mono(2-ethylhexyl) phthalate; MEOHP: mono(2-ethyl-5-oxohexyl) phthalate; MEP: monoethyl phthalate; MEPB: methyl paraben; MiBP; MMP: momomethyl phthalate; MnBP: mono-n-butyl phthalate; MSEL: Mullen Scales of Early Learning; NEPSY-II: A Developmental NEuroPSYchological Assessment; OC: organochlorine; OP: organophosphate; OR: odds ratio; p,p’-DDE: p,p’-dichlorodiphenyldichloroethylene; p,p’-DDT: p,p’-dichlorodiphenyltrichloroetane; PA: phthalic acid; PBA: phenoxybenzoic acid; PBB: polybrominated biphenyl; PBDE: prolibrominated diphenyl ether; PCB: polychlorinated biphenyls; PCDD/F: polychlorinated dibenzo-p-dioxins; PCDD/Fs: polychlorinated dibenzo-p-dioxins and dibenzofurans; PDP: Pervasive Developmental Problem scale; PFAS: perfluoroalkyl substances; PFDA: perfluorodecanoic acid; PFHxS: perfluorohexane sulfonic acid; PFHpS: perfluoroheptasulfonic acid; PFNA: perfluorononanoic acid; PFOA: perfluorooctanoic acid; PFOS: perfluorooctane sulfonate; PRPB: propyl paraben; RR: relative risk; RRR: relative risk ratio; SD: standard deviation; SDQ: Strength and Difficulties Questionnaire; SQ: Systemizing Quotient; SRS: Social Responsiveness Scale; TCPy: 3,5,6-trichloro-2-pyridinol; TCS: triclosan.
