# Supplementary material for: Early-life exposure to endocrine-disrupting chemicals and autistic traits in childhood and adolescence: a systematic review of epidemiological studies
Source: Front Endocrinol (Lausanne). 2023 Jun 9;14:1184546. doi: 10.3389/fendo.2023.1184546 (PMC10289191; doi:10.3389/fendo.2023.1184546)
Supplement: Supplementary file 4 [file Table_4.docx]

**Supplementary Table 4.** Excluded articles and reasons for exclusion (n = 20).

| Authors, Year | Reason for exclusion |
| --- | --- |
| Al-Saleh et al., 2021 (1) | 1 |
| Cheslack-Postava et al., 2022 (2) | 1 |
| Christian et al., 2019 (3) | 2 |
| Cock et al., 2016 (4) | 3 |
| Eskenazi et al., 2007 (5) | 1 |
| Eskenazi et al., 2010 (6) | 1 |
| Furlong et al., 2017 (7) | 1 |
| Joyce et al., 2021 (8) | 2 |
| Keil et al., 2014 (9) | 2 |
| Palmer et al., 2015 (10) | 1 |
| Rauh et al., 2006 (11) | 1 |
| Ritz et al., 2020 (12) | 3 |
| Roberts et al., 2007 (13) | 2 |
| Roberts et al., 2013 (14) | 3 |
| Roberts et al., 2013 (15) | 2 |
| Schmidt et al., 2017 (16) | 2 |
| van Ehrenstein et al., 2019 (17) | 2 |
| Windham et al., 2006 (18) | 2 |
| Wu et al., 2022 (19) | 2 |
| Yong et al., 2021 (20) | 2 |

1. Inappropriate outcome (overall developmental delay, intellectual deficiency, attention deficit and hyperactivity disorder)

2. Inappropriate exposure

3. Inappropriate design

**References**

(1) Al-Saleh I, Elkhatib R, Alrushud N, Alnuwaysir H, Alnemer M, Aldhalaan H, et al. Potential health risks of maternal phthalate exposure during the first trimester - the saudi early autism and environment study (seaes). *Environmental Research* (2021) 195(doi:10.1016/j.envres.2021.110882

(2) Cheslack-Postava K, Rantakokko P, Kiviranta H, Hinkka-Yli-Salomäki S, Surcel HM, Vivio N, et al. Maternal serum persistent organic pollutant exposure and offspring diagnosed adhd in a national birth cohort. *Environmental Research* (2022) 212(doi:10.1016/j.envres.2022.113145

(3) Christian MKA, Samms-Vaughan M, Lee MJ, Bressler J, Hessabi M, Grove ML, et al. Maternal exposures associated with autism spectrum disorder in jamaican children. *Journal of Autism and Developmental Disorders* (2018) 48(8):2766-78. doi:10.1007/s10803-018-3537-6

(4) de Cock M, Quaak I, Sugeng EJ, Legler J, and van de Bor M. Linking edcs in maternal nutrition to child health (linc study) - protocol for prospective cohort to study early life exposure to environmental chemicals and child health. *Bmc Public Health* (2016) 16(doi:10.1186/s12889-016-2820-8

(5) Eskenazi B, Marks AR, Bradman A, Harley K, Barr DB, Johnson C, et al. Organophosphate pesticide exposure and neurodevelopment in young mexican-american children. *Environmental Health Perspectives* (2007) 115(5):792-8. doi:10.1289/ehp.9828

(6) Eskenazi B, Huen K, Marks A, Harley KG, Bradman A, Barr DB, et al. Pon1 and neurodevelopment in children from the chamacos study exposed to organophosphate pesticides in utero. *Environmental Health Perspectives* (2010) 118(12):1775-81. doi:10.1289/ehp.1002234

(7) Furlong MA, Barr DB, Wolff MS, and Engel SM. Prenatal exposure to pyrethroid pesticides and childhood behavior and executive functioning. *Neurotoxicology* (2017) 62(231-8. doi:10.1016/j.neuro.2017.08.005

(8) Joyce EE, Chavarro JE, Rando J, Song AY, Croen LA, Fallin MD, et al. Prenatal exposure to pesticide residues in the diet in association with child autism-related traits: Results from the earli study. *Autism Res* (2022) 15(5):957-70. doi:10.1002/aur.2698

(9) Keil AP, Daniels JL, and Hertz-Picciotto I. Autism spectrum disorder, flea and tick medication, and adjustments for exposure misclassification: The charge (childhood autism risks from genetics and environment) case-control study. *Environ Health* (2014) 13(1):3. doi:10.1186/1476-069x-13-3

(10) Palmer RF, Heilbrun L, Camann D, Yau A, Schultz S, Elisco V, et al. Organic compounds detected in deciduous teeth: A replication study from children with autism in two samples. *J Environ Public Health* (2015) 2015(862414. doi:10.1155/2015/862414

(11) Rauh VA, Garfinkel R, Perera FP, Andrews HF, Hoepner L, Barr DB, et al. Impact of prenatal chlorpyrifos exposure on neurodevelopment in the first 3 years of life among inner-city children. *Pediatrics* (2006) 118(6):e1845-e59. doi:10.1542/peds.2006-0338

(12) Ritz B, Yan Q, Uppal K, Liew Z, Cui X, Ling C, et al. Untargeted metabolomics screen of mid-pregnancy maternal serum and autism in offspring. *Autism Res* (2020) 13(8):1258-69. doi:10.1002/aur.2311

(13) Roberts EM, English PB, Grether JK, Windham GC, Somberg L, and Wolff C. Maternal residence near agricultural pesticide applications and autism spectrum disorders among children in the california central valley. *Environ Health Perspect* (2007) 115(10):1482-9. doi:10.1289/ehp.10168

(14) Roberts EM, and English PB. Bayesian modeling of time-dependent vulnerability to environmental hazards: An example using autism and pesticide data. *Stat Med* (2013) 32(13):2308-19. doi:10.1002/sim.5600

(15) Roberts AL, Lyall K, Hart JE, Laden F, Just AC, Bobb JF, et al. Perinatal air pollutant exposures and autism spectrum disorder in the children of nurses' health study ii participants. *Environmental Health Perspectives* (2013) 121(8):978-84. doi:10.1289/ehp.1206187

(16) Schmidt RJ, Kogan V, Shelton JF, Delwiche L, Hansen RL, Ozonoff S, et al. Combined prenatal pesticide exposure and folic acid intake in relation to autism spectrum disorder. *Environ Health Perspect* (2017) 125(9):097007. doi:10.1289/ehp604

(17) von E. Prenatal and infant exposure to ambient pesticides and autism spectrum disorder in children: Population based case-control study (vol 364, l962, 2019). *Bmj-British Medical Journal* (2019) 365(doi:10.1136/bmj.l4032

(18) Windham GC, Zhang L, Gunier R, Croen LA, and Grether JK. Autism spectrum disorders in relation to distribution of hazardous air pollutants in the san francisco bay area. *Environ Health Perspect* (2006) 114(9):1438-44. doi:10.1289/ehp.9120

(19) Wu Q, Yang T, Chen L, Dai Y, Wei H, Jia F, et al. Early life exposure to triclosan from antimicrobial daily necessities may increase the potential risk of autism spectrum disorder: A multicenter study in china. *Ecotoxicol Environ Saf* (2022) 247(114197. doi:10.1016/j.ecoenv.2022.114197

(20) Yong Z, Dou Y, Gao Y, Xu X, Xiao Y, Zhu H, et al. Prenatal, perinatal, and postnatal factors associated with autism spectrum disorder cases in xuzhou, china. *Transl Pediatr* (2021) 10(3):635-46. doi:10.21037/tp-21-54
