# Supplementary material for: Early-life exposure to endocrine-disrupting chemicals and autistic traits in childhood and adolescence: a systematic review of epidemiological studies
Source: Front Endocrinol (Lausanne). 2023 Jun 9;14:1184546. doi: 10.3389/fendo.2023.1184546 (PMC10289191; doi:10.3389/fendo.2023.1184546)
Supplement: Supplementary file 5 [file Table_5.docx]

**Supplementary Table 5.** Risk of bias summary, assessed by Joanna Briggs Institute Critical Appraisal Checklist for Case-Control Studies: author’s judgments for each included study.

| Authors, year | Q1 | Q2 | Q3 | Q4 | Q5 | Q6 | Q7 | Q8 | Q9 | Q10 | Total | Risk of bias |
| --- | --- | --- | --- | --- | --- | --- | --- | --- | --- | --- | --- | --- |
| Brown et al., 2018 | Y | Y | Y | Y | Y | Y | Y | Y | Y | Y | 10 | LOW |
| Hamra et al., 2019 | U | U | Y | Y | Y | Y | Y | Y | Y | Y | 8 | LOW |
| Liew et al., 2015 | Y | Y | Y | Y | Y | Y | Y | Y | Y | Y | 10 | LOW |
| Long et al., 2019 | Y | Y | Y | Y | Y | Y | Y | Y | U | Y | 9 | LOW |
| Lyall et al., 2017 (1) | Y | Y | Y | Y | Y | Y | Y | Y | Y | Y | 10 | LOW |
| Lyall et al., 2017 (2) | Y | Y | Y | Y | Y | Y | Y | Y | Y | Y | 10 | LOW |

Legend: Y= Yes; N= No; U= Unclear; NA= Not applicable

Q1- Were the groups comparable other than the presence of disease in cases or the absence of disease in controls?

Q2- Were cases and controls matched appropriately?

Q3- Were the same criteria used for identification of cases and controls?

Q4- Was exposure measured in a standard, valid and reliable way?

Q5- Was exposure measured in the same way for cases and controls?

Q6- Were confounding factors identified?

Q7- Were strategies to deal with confounding factors stated?

Q8- Were outcomes assessed in a standard, valid and reliable way for cases and controls?

Q9- Was the exposure period of interest long enough to be meaningful?

Q10- Was appropriate statistical analysis used?

Total= ΣY/Applicable Items (the Not Applicable (NA) items were excluded from the sum).

Risk of bias was categorized as high when the study reaches up to 49% score “yes”, moderate when the study reached 50% to 69% score “yes”, and low when the study reached more than 70% score “yes

**Supplementary Table 4.** Risk of bias summary, assessed by Joanna Briggs Institute Critical Appraisal Checklist Cohort Studies: author’s judgments for each included study.

| Authors, year | Q1 | Q2 | Q3 | Q4 | Q5 | Q6 | Q7 | Q8 | Q9 | Q10 | Q11 | Total | Risk of bias |
| --- | --- | --- | --- | --- | --- | --- | --- | --- | --- | --- | --- | --- | --- |
| Alempi et al., 2021 | Y | Y | Y | Y | Y | U | Y | Y | Y | U | Y | 9 | LOW |
| Barkoski et al., 2019 | Y | Y | Y | Y | Y | U | Y | Y | U | U | Y | 8 | LOW |
| Barkoski et al., 2021 | Y | Y | Y | Y | Y | U | Y | Y | U | U | Y | 8 | LOW |
| Bernardo et al., 2019 | Y | Y | Y | Y | Y | U | Y | Y | U | U | Y | 8 | LOW |
| Braun et al., 2014 | Y | Y | Y | Y | Y | U | Y | Y | U | U | Y | 8 | LOW |
| Haggerty et al., 2021 | Y | Y | Y | Y | Y | U | Y | U | U | U | Y | 7 | MODERATE |
| Hansen et al., 2021 | Y | Y | Y | Y | Y | U | Y | Y | N | N | Y | 8 | LOW |
| Lim et al., 2017 | Y | Y | Y | Y | Y | U | Y | Y | N | N | Y | 8 | LOW |
| Lizé et al., 2022 | Y | Y | Y | Y | Y | U | Y | Y | N | N | Y | 8 | LOW |
| Millenson et al., 2017 | Y | Y | Y | Y | Y | U | Y | Y | N | N | Y | 8 | LOW |
| Miodonovic et al., 2011 | Y | Y | Y | Y | Y | U | Y | Y | Y | N | Y | 9 | LOW |
| Nowack et al., 2015 | Y | Y | Y | Y | Y | U | Y | Y | Y | N | Y | 9 | LOW |
| Ou et al., 2021 | Y | Y | Y | Y | Y | U | Y | Y | N | N | Y | 8 | LOW |
| Oulhote et al., 2016 | Y | Y | Y | Y | Y | U | Y | Y | N | N | Y | 8 | LOW |
| Oulhote et al., 2020 | Y | Y | Y | Y | Y | U | Y | Y | Y | N | Y | 9 | LOW |
| Patti et al., 2021 | Y | Y | Y | Y | Y | U | Y | Y | Y | N | Y | 9 | LOW |
| Phillippat et al., 2018 | Y | Y | Y | Y | Y | U | Y | Y | Y | N | Y | 9 | LOW |
| Sagiv et al., 2018 | Y | Y | Y | Y | Y | U | Y | Y | Y | N | Y | 9 | LOW |
| Shin et al., 2018 | Y | Y | Y | Y | Y | U | Y | Y | N | N | Y | 8 | LOW |
| Van den Dries et al., 2019 | Y | Y | Y | Y | Y | U | Y | Y | Y | N | Y | 9 | LOW |
| Van den Dries et al., 2021 | Y | Y | Y | Y | Y | U | Y | Y | Y | N | Y | 9 | LOW |

Legend: Y= Yes; N= No; U= Unclear; NA= Not applicable

Q1- Were the two groups similar and recruited from the same population?

Q2- Were the exposures measured similarly to assign people to both exposed and unexposed groups?

Q3- Was the exposure measured in a valid and reliable way?

Q4- Were confounding factors identified?

Q5- Were strategies to deal with confounding factors stated?

Q6- Were the groups/participants free of the outcome at the start of the study (or at the moment of exposure)?

Q7- Were the outcomes measured in a valid and reliable way?

Q8- Was the follow up time reported and sufficient to be long enough for outcomes to occur?

Q9- Was follow up complete, and if not, were the reasons to loss to follow up described and explored?

Q10- Were strategies to address incomplete follow up utilized?

Q11- Was appropriate statistical analysis used?

Total= ΣY/Applicable Items (the Not Applicable (NA) items were excluded from the sum).

Risk of bias was categorized as high when the study reaches up to 49% score ‘yes’, moderate when the study reached 50% to 69% score ‘yes’, and low when the study reached more than 70% score ‘yes’.
